# Chromatin state analysis of the barley epigenome reveals a higher‐order structure defined by H3K27me1 and H3K27me3 abundance

**DOI:** 10.1111/tpj.12963

**Published:** 2015-09-09

**Authors:** Katie Baker, Taniya Dhillon, Isabelle Colas, Nicola Cook, Iain Milne, Linda Milne, Micha Bayer, Andrew J. Flavell

**Affiliations:** ^1^ University of Dundee at JHIInvergowrieDundeeDD2 5DAUK; ^2^ James Hutton InstituteInvergowrieDundeeDD2 5DAUK; ^3^ Present address: University of St AndrewsSt AndrewsKY16 9THUK

**Keywords:** epigenomics, heterochromatin, pericentromeric, chromatin immunoprecipitation next‐generation sequencing, histone modification, barley, *Hordeum vulgare*, PRJEB8068

## Abstract

Combinations of histones carrying different covalent modifications are a major component of epigenetic variation. We have mapped nine modified histones in the barley seedling epigenome by chromatin immunoprecipitation next‐generation sequencing (ChIP‐seq). The chromosomal distributions of the modifications group them into four different classes, and members of a given class also tend to coincide at the local DNA level, suggesting that global distribution patterns reflect local epigenetic environments. We used this peak sharing to define 10 chromatin states representing local epigenetic environments in the barley genome. Five states map mainly to genes and five to intergenic regions. Two genic states involving H3K36me3 are preferentially associated with constitutive gene expression, while an H3K27me3‐containing genic state is associated with differentially expressed genes. The 10 states display striking distribution patterns that divide barley chromosomes into three distinct global environments. First, telomere‐proximal regions contain high densities of H3K27me3 covering both genes and intergenic DNA, together with very low levels of the repressive H3K27me1 modification. Flanking these are gene‐rich interior regions that are rich in active chromatin states and have greatly decreased levels of H3K27me3 and increasing amounts of H3K27me1 and H3K9me2. Lastly, H3K27me3‐depleted pericentromeric regions contain gene islands with active chromatin states separated by extensive retrotransposon‐rich regions that are associated with abundant H3K27me1 and H3K9me2 modifications. We propose an epigenomic framework for barley whereby intergenic H3K27me3 specifies facultative heterochromatin in the telomere‐proximal regions and H3K27me1 is diagnostic for constitutive heterochromatin elsewhere in the barley genome.

## Significance Statement

Triticeae cereals are among the most important crops worldwide yet their epigenomics are largely unstudied. Here we have used ChIP‐Seq to explore the epigenome of barley, identifying and mapping chromatin states of nine modified histones at both gene and genome levels.

## Introduction

Reversible covalent modification of DNA and its associated chromatin proteins defines the epigenome. Nucleosomes are the major protein constituents of chromatin, and modification of their component histones H2A, H2B, H3 and H4 modulates nucleosomal properties in a complex and incompletely understood manner to regulate chromosomal packaging, replication, recombination and expression (Berger, 2007; Dorn and Cook, 2011). Numerous histone modifications comprising this ‘histone code’ (Jenuwein and Allis, 2001) have been described, and the most widely studied involve addition of methyl or acetyl groups to lysine residues of histones H3 and H4 (Kouzarides, 2007). Active chromatin modifications, such as acetylation at H3K9 and trimethylation at H3K4, are associated with the start sites of transcribed genes, whereas repressive marks, including dimethylation or trimethylation at H3K9 in plants or animals, respectively, typify the highly compacted constitutive heterochromatic state (Lippman *et al*., 2004).

An important class of histone modifications involves H3K27 methylation. H3K27me3 plays a central role in the establishment of facultative (reversible) heterochromatin in animals, Arabidopsis and maize (Schuettengruber *et al*., 2007; Lafos *et al*., 2011; Makarevitch *et al*., 2013), regulating multiple developmentally regulated genes in Arabidopsis (Holec and Berger, 2012) and showing tissue‐specific variation in genomic distribution in maize (Makarevitch *et al*., 2013). Conversely, H3K27me1 is selectively enriched in the constitutive heterochromatin of both animals and Arabidopsis (Peters *et al*., 2003; Jacob *et al*., 2010).

The chromosomal locations of heterochromatic regions vary between species, but they are commonly seen in the pericentromeric (PC) regions surrounding centromeres. Plant heterochromatin tends to display low recombination rates, low gene content and high DNA repeat density, with many of the repeats composed of transposable elements (TEs). The low‐recombining (LR) PC regions are huge in the major food crop cereal grasses, comprising 50% or more of the total genome of barley for example (IBGSC 2012; Baker *et al*., 2014). For cereals there tend to be high levels of repressive epigenetic marks in the PC regions, but clear evidence also exists for such marks across the genome (Houben *et al*., 2003; Shi and Dawe, 2006; Carchilan *et al*., 2007; Gent *et al*., 2012; Higgins *et al*., 2012) and in maize no chromatin modification so far tested clearly differentiates pericentromeres, centromeres and chromosome arms (Gent *et al*., 2012). The PC regions of cereals carry high densities of long terminal repeat (LTR) retrotransposon insertions (Paterson *et al*., 2009, Schnable *et al*., 2009; IBGSC 2012), consistent with the model first proposed for Arabidopsis that heterochromatin is defined at the local level by TEs (Lippman *et al*., 2004) and may be present anywhere in the genome. Major differences in histone modification are apparent between genes and TEs in all plant species explored to date (Li *et al*., 2008; Wang *et al*., 2009; He *et al*., 2010).

In animals the positioning of heterochromatin near genes can lead to suppressed gene expression (Jost *et al*., 2012) but this does not seem to be the case in plants. In *Arabidopsis thaliana*, genes surrounded by heterochromatin are insulated from heterochromatic silencing (Lippman *et al*., 2004), suggesting that there are mechanisms to prevent heterochromatin from repressing adjacent gene expression. Similarly, in barley average mRNA levels per gene are similar in the PC region to the rest of the genome (Baker *et al*., 2014). In maize, TEs adjacent to genes are depleted for H3K9me2, have euchromatic signatures and are silenced by small interfering RNAs (Gent *et al*., 2014). Conversely, some genes carry H3K9me2 modification, particularly if they bear intronic TE insertions (West *et al*., 2014). For barley the local chromatin environments of the genes in any genomic region is largely unknown.

Epigenetic state can be investigated by chromatin immunoprecipitation (ChIP) using antibodies raised against modified histones. Immunoselected DNA can be assayed either by quantitative PCR (for individual genes) or at a genome‐wide level by hybridization to tiling arrays or by next‐generation sequencing (NGS) (the ChIP‐seq technique). All three approaches have been applied to plants. The *A. thaliana* epigenome has been extensively studied and the distributions of a wide range of covalent modifications to histones and DNA have been characterized (Lippman *et al*., 2004; Roudier *et al*., 2011; Sequeira‐Mendes *et al*., 2014). These studies have shown that histone modifications display individual distribution patterns with regard to genes and TEs and these patterns vary depending upon gene activity.

Analyses of peak sharing along the genomes of *A. thaliana* and several animal species have shown that certain combinations of histone modification are frequent, leading to the model that a genome can be subdivided into regions carrying characteristic combinations of epigenetic marks that define corresponding chromatin states (Ernst and Kellis, 2010; Roudier *et al*., 2011; Sequeira‐Mendes *et al*., 2014). Active chromatin states can be specific to small sub‐regions of genes, such as transcriptional start sites (TSSs), while repressive states can cover extensive intergenic regions rich in TEs.

The epigenomes of other plant species have received less attention than that of *A. thaliana*, with most studies being focused on cereals, particularly maize and rice (Shi and Dawe, 2006; Li *et al*., 2008; Wang *et al*., 2009; He *et al*., 2010; Gent *et al*., 2012; Makarevitch *et al*., 2013), but with very little attention being given to wheat or barley. Barley is the fourth most important crop cereal worldwide, an inbreeding diploid species and a model for genomic research in other important Triticeae crops, particularly wheat. Large genomes come with specific challenges for genomics‐based approaches, mainly due to their large expanses of repetitive DNA that lead to a fragmented reference genome. The ChIP‐seq technique is an effective tool for analyzing the gene space of the epigenomes of plants with large genomes because DNA libraries immunoselected for gene‐associated marks are not much larger than those for plants with small genomes. However, modifications associated with repetitive regions (around 75% of the barley genome) are problematical because NGS reads map to multiple loci and their true origin cannot be discerned. Nevertheless, a global description of the epigenetic marks for intergenic regions is achievable.

In the present study we have explored the epigenomic landscape of barley seedlings using ChIP‐seq and report the distributions for nine modified histones. We have used these data to study the relationships between histone modification, the epigenetic environment at both gene and genome level and the interplay between chromatin state and gene expression for barley.

## Results

### Epigenomic profiles for nine modified histone marks

We performed ChIP‐seq on whole barley seedlings using antibodies specific for H3K4me2, H3K4me3, H3K9me2, H3K9me3, H3K27me1, H3K27me2, H3K27me3, H3K36me3 and H3K56ac plus unmodified H3. The resulting NGS reads were mapped to the barley cv. Morex genome assembly (IBGSC 2012) and peaks of histone enrichment were called (see Experimental Procedures and Table S1 in Supporting Information).

**Figure 1:**
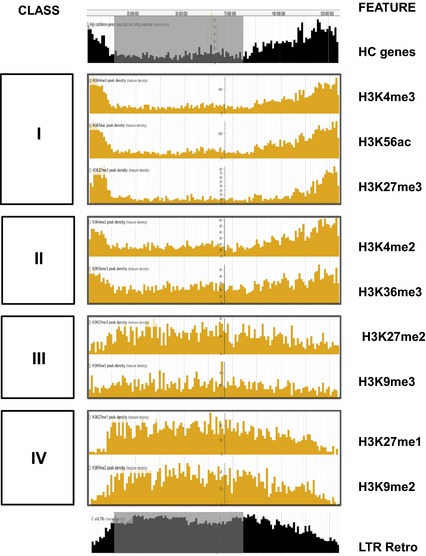
Densities of assigned peaks for histone modifications across barley chromosome 1H. Local peak densities (peaks per 1‐Mbp bin) for the nine histone modifications studied here are plotted against pseudophysical position on barley chromosome 1H in JB rowse (see Experimental Procedures). Distribution Classes I–IV are described in the text. The corresponding densities of high‐confidence (HC) genes and long terminal repeat retrotransposons (LTR Retro) are shown in black and the location of the low‐recombining pericentromeric region (Baker *et al*., 2014) is in gray.

To visualize the chromosomal distributions of the peak densities and the other genome‐scale features described here, we created a web‐based genome browser containing these data (see Experimental Procedures). The chromosomal distributions of peak densities, together with high‐confidence (HC) genes (IBGSC 2012), LTR retrotransposons and the LR‐PC region are shown in Figure 1 for chromosome 1H and data for all chromosomes are given in Figure S1. Different distribution profiles are apparent, and we grouped these into four classes. Class I modifications (H3K4me3, H3K56ac, H3K27me3) show high peak densities towards the telomeres and low densities in the LR‐PC region, which is shaded in grey in Figure 1 (Baker *et al*., 2014). This distribution pattern closely follows the profile of the high‐confidence (HC) genes of barley (Figure 1; IBGSC 2012) and supports a role for these modifications in the genic chromatin environment of barley. Note that the profile for H3K27me3 shows a particularly strong bias against the PC region and tighter association with the telomeres than the other two Class I modifications. We will return to this important point later. Class II modifications (H3K4me2 and H3K36me3) also show high peak densities in the gene‐rich telomeric regions but they are more frequent in the LR‐PC region than Class I modifications. Class III marks (H3K27me2 and H3K9me3) show rather even but patchy chromosomal distributions.

Lastly, the Class IV histone modifications H3K27me1 and H3K9me2 are depleted in the telomere‐proximal regions and enriched in the interior of the chromosome. This demarcation is particularly acute for H3K27me1, whose distribution is inverse to that of H3K27me3. H3K9me2 closely follows LTR retrotransposon density, suggesting a similar role for this modification to that in Arabidopsis, where it is a mark for constitutive heterochromatin and involved in epigenetic repression of LTR retrotransposon activity (Peters *et al*., 2003; Jacob *et al*., 2010).

### Gene profiles for modified histone marks

**Figure 2:**
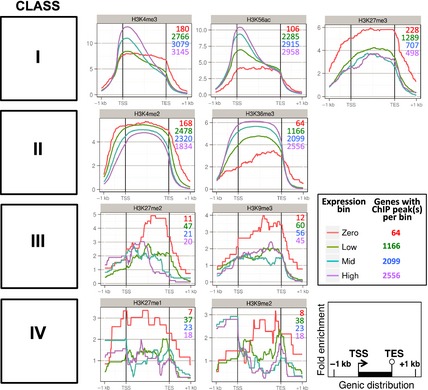
Histone modification profiles across barley genes. Mean fold‐change enrichments in chromatin immunoprecipitation next‐generation sequencing (ChIP‐seq) peaks for each histone modification, relative to the input DNA control, are shown. Histone modifications are ordered by chromosomal distribution class (see Figure 1). Enrichment values are averaged across all barley high‐confidence genes after binning into four expression levels (see Experimental Procedures), which are indicated by different colored lines as shown. The total numbers of genes with corresponding ChIP‐seq peaks in the four expression bins are also shown and color‐coded similarly. Gene lengths are normalized against each other, introns have been removed and regions from 1 kb upstream of the transcription start site (TSS) to 1 kb downstream of the transcription end site (TES) are included.

We next related our ChIP‐Seq peaks to the HC genes of barley (Figure 2; IBGSC 2012). Two Class I modifications, H3K4me3 and H3K56ac, show strong ChIP‐seq enrichment at the transcription start site (TSS), with the magnitude of the enrichment being positively correlated with the corresponding gene expression level (genes were grouped into four expression‐level bins ranging from zero to high; see Experimental Procedures). In contrast, H3K27me3 displays broad gene coverage that is largely unresponsive to the gene expression level and is enhanced in unexpressed genes. Class II modifications also show broad distributions across gene bodies, with H3K4me2 displaying a slight reduction in peak enrichment with increasing gene expression, whereas H3K36me3 shows a strong positive correlation with gene expression. All five Class I–II modifications are strongly enriched in genes, with peak enrichment levels between five and fifteen‐fold higher than the background (input) signal (see Experimental Procedures). These data are consistent with the increased genomic signal for these five modifications in the gene‐rich regions near the telomeres (Figure 1) and the conclusion that they all play a role in the genic chromatin environment of barley.

The genic distribution profiles of the four remaining histone modifications are quite similar to each other, with little or no enrichment in expressed genes and higher enrichment in unexpressed genes. There are very few gene‐associated peaks for any of these modifications (Figure 2, Table S2), consistent with their sparse distribution in the gene‐rich chromosomal regions (Figure 1). We conclude that Class III and IV histone modifications are at best only weakly associated with a small number of barley genes.

### Sharing of modified histone marks and chromatin state analysis of the barley epigenome

**Figure 3:**
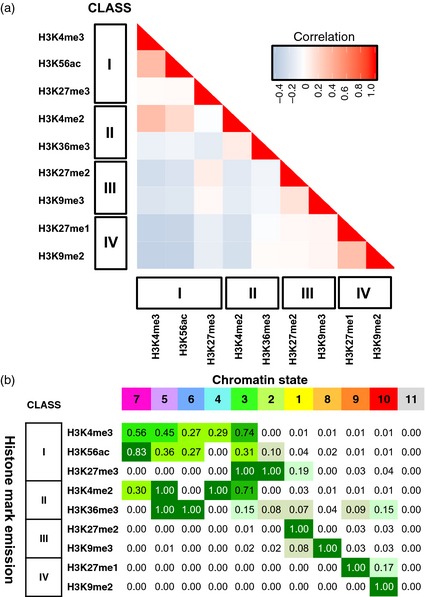
Analysis of peak sharing between modified histones and definition of chromatin states for barley. (a) Correlation analysis of peak sharing. Modified histones are ordered in both *x* and *y* axes by chromosome distribution class (Figure 1). The peak sharing level is colour coded as indicated in the key. (b) State emissions for an 11‐state model of the barley epigenome derived from peak sharing among the nine histone modifications in this study. States (top row) are ordered from left to right and color‐coded correspondingly (magenta to red) by decreasing involvement of their component histone modifications with the active genic environment (see Figure 2). Histone modifications (left column) are ordered by genomic distribution class (Figure 1). Each state emission (cell value) represents the probability of involvement of a chromatin mark in the corresponding state. State 11 is a zero state (no associated histone peaks).

Particular combinations of modified histones associate with the different genic and genomic features to regulate the functioning of the epigenome (Jenuwein and Allis, 2001; Berger, 2007; Ernst and Kellis, 2010; Dorn and Cook, 2011; Roudier *et al*., 2011; Sequeira‐Mendes *et al*., 2014). We therefore used correlation analysis to explore peak sharing between barley histone modifications (Figure 3a). All of the modifications apart from H3K27me3 display positive peak correlations (pink sectors) within distribution classes. In addition there is considerable peak sharing between Classes I and II, which are all gene‐associated marks (Figure 2) with rather similar genome distributions (Figure 1). Thus, genomic distributions for these eight chromatin marks (Class designation, Figure 1) are related to their peak overlaps at the local level and vice versa.

The sole exception to this picture, H3K27me3, shows positive peak correlation with the Class III modifications H3K27me2 and H3K9me3 but is virtually neutral with respect to its other Class I members, despite its strong overlaps with the latter at the chromosome and gene levels (Figures 1 and 2). This suggests that H3K27me3 either resides on a different gene set from the other gene‐associated chromatin marks or it shares gene targets with them but also has many other peak locations.

Combinations of epigenetic marks can be grouped into chromatin states, which describe the most probable combinations of shared chromatin peaks that define local epigenetic environments. We used chromHMM (Ernst and Kellis, 2012) (Table S3, Figure S2 and Experimental Procedures) to find an 11‐state model that represents the 10 most frequent combinations of modifications in our dataset, together with a zero state containing no modified histone peaks. In Figure 3(b) we have ordered and color‐coded these states by their decreasing involvements with active chromatin histone marks. States 7, 5, 6 and 4 are dominated by active chromatin histone marks from Classes I and II and state 3 combines these modifications with the repressive H3K27me3 mark (Lafos *et al*., 2011). The remaining five states are each dominated by a different repressive modification from Classes I, III and IV.

**Figure 4:**
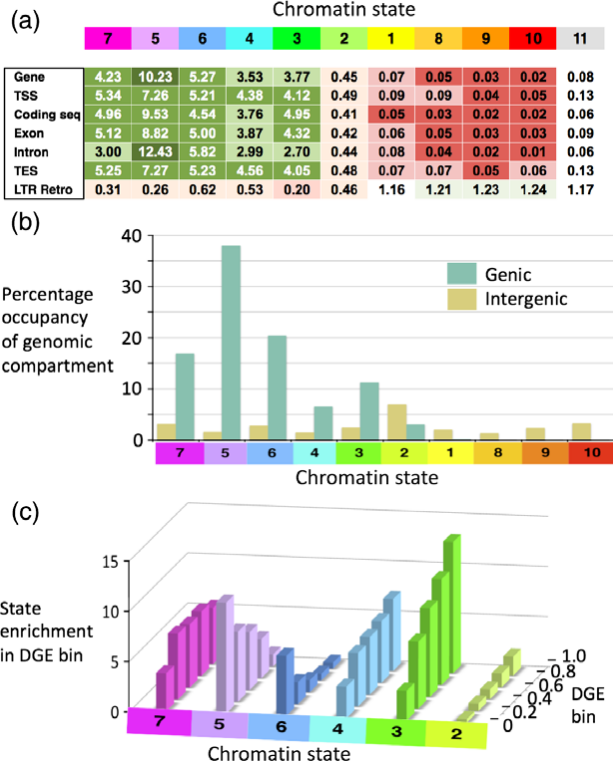
Biological properties of barley chromatin states. (a) Enrichments of chromatin states in gene features and long terminal repeat (LTR) retrotransposons. Fold enrichments for barley chromatin states in annotated genomic features are indicated (see Results). Cell values are color‐coded from dark green (highly enriched) through white (no enrichment) to dark red (strong negative enrichment). TSS, transcription start site; CDS, protein‐coding sequence; TES, transcription end site; LTR Retro, LTR retrotransposon. (b) Genic and intergenic occupancies of chromatin states. Total percentage occupancies for each state in total genic (green) and intergenic (khaki) spaces are shown. (c) Relationships between chromatin state enrichment and differential gene expression (DGE). Genes are binned by five DGE levels from low (<0.2) to high (>0.8) differential expression in seedling root versus seeding shoot. Fold enrichments (see Results) for the six gene‐associated states in each DGE bin (see panels a and b) are shown.

To explore the genomic and genic properties of these states we investigated sequence annotations associated with them (Figure 4a). Each value in the figure shows the frequency for an annotated genomic feature in the corresponding state, relative to its average genomic frequency. Thus, the term ‘intron’ features roughly 12.43 times more frequently in state 5 annotations (lilac) than it does in all annotations. These frequencies, together with the state emission data (Figure 3b), allow us to assign putative properties to the states. States 3–7 are all strongly genic states, with strong emissions from active chromatin marks of Classes I and II (Figure 3b) and weak overlaps with LTR retrotransposons (0.20–0.63). State 7, is a candidate TSS state, with the highest annotation frequency for this region (5.34) and strong emission weightings from H3K56ac and H3K4me3, which peak around the TSS (Figure 2). State 5 has a particularly high frequency in introns (12.43), whereas states 6 and 4 both show a broad, non‐specific distribution across gene bodies and are mainly distinguished by their involvement with either H3K36me3 (State 6) or H3K4me2 (state 4). State 3 superimposes the repressive mark H3K27me3 upon the other four gene‐associated marks studied here (Figure 3b), suggesting a role in tissue‐specific, differentially regulated gene expression. State 2, the other H3K27me3‐dominated state (Figure 3b), transitions between the genic and intergenic environments, with frequencies of 0.4–0.5 for both genes and LTR retroransposons (Figure 4a). The final four states (states 1, 8, 9, 10) all show low frequencies for gene annotations (<0.1) and higher frequencies (>1) for LTR retrotransposons.

We also looked at the distribution of states between total genic and intergenic spaces via their annotations (Figure 4b). Almost all annotated gene space (93%) is occupied by states 3–7. State 2 again occupies both genic (3%) and intergenic (7%) spaces but as the latter is 20‐fold greater than the genic space in barley (IBGSC 2012), state 2 is overwhelmingly intergenic. The four repressive chromatin states (1,8,9,10) are almost absent from the genic compartment.

Lastly, we investigated the relationship between state and differential gene expression (DGE) between root and seedling tissue (see Experimental Procedures). Genes were clustered into five discrete DGE bins, from 0 (no differential expression) to 1 (high differential expression), using a *P*‐ranking approach (Yu *et al*., 2010). Enrichments in these bins for the six chromatin states with genic involvement are shown in Figure 4(c). Large differences in DGE are visible between states. In particular, State 3 enrichment shows a strong positive relationship with DGE, states 2 and 4 show weaker positive trends and states 5 and 6 show strong negative relationships. We conclude that state 3 plays a major role in DGE in the developing barley seedling, states 2 and 4 have weaker roles, states 5 and 6 are involved in constitutive gene expression and state 7 plays little or no role in DGE.

### Epigenomic analysis of barley chromatin states reveals an unexpected higher‐order structure in barley chromosomes

**Figure 5:**
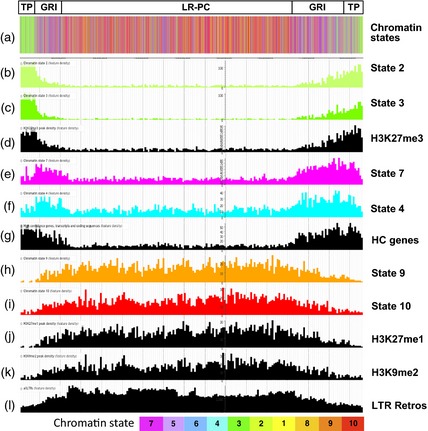
Higher‐order epigenomic structure of barley chromosome 2H. Telomere‐proximal (TP), gene‐rich interior (GRI) and low‐recombining pericentromeric (LR‐PC) regions are boxed. Chromatin states are color coded as shown in the key at the bottom of the figure and other features are in black. (a) All chromatin states; (b)–(l) densities in 1‐Mbp bins for individual chromatin states (b, c, e, f, h, i), modified histone peaks (d, j, k), high‐confidence (HC) genes (g) and LTR retrotransposons (l).

The distribution of the chromatin states on barley chromosome 2H is shown in Figure 5(a) and data for all chromosomes are in Figure S3. This reveals striking high‐order localization patterns for particular chromatin states on most chromosome arms. States 2 and 3 localize strongly to telomere‐proximal (TP) regions (Figure 5a–c). Adjacent to these are gene‐rich interior (GRI) regions with high densities of gene‐associated states such as 7 and 4 (Figure 5a, e, f). Lastly, the gene‐poor LR‐PC region is dominated by repressive states such as states 9 and 10 (Figure 5a, h, i).

These chromosomal state distributions can be understood in terms of the corresponding distributions of the genes and LTR retrotransposons, together with the histone modifications that contribute to the states. States 2 and 3, which define the TP region, are dominated by H3K27me3 (Figure 3b) and these states follow the chromosomal distribution of this mark (Figure 5b–d). States 7 and 4 are gene‐associated and thus follow the distribution of genes in the GRI (Figure 5e–g). States 7 and 4 are depleted relative to genes in the TP region (Figure 5e–g) because the prevalent genic H3K27me3 mark in this region switches these states to state 3 (Figures 3b and 5c). Lastly, the extensive interior LR‐PC region is enriched for states 9 and 10 (Figure 5h–l), which are dominated by the heterochromatic marks H3K27me1 and H3K9me2, respectively (Figure 3b). These two states associate with LTR retrotransposons (Figures 4a and 5l) and are depleted in the TP region (Figure 5h–k). In particular, H3K27me1 shows an inverse distribution to H3K27me3 (Figure 5d, j) that defines the boundaries between the TP and GRI regions.

### Local epigenetic environment in barley epigenomic regions

**Figure 6:**
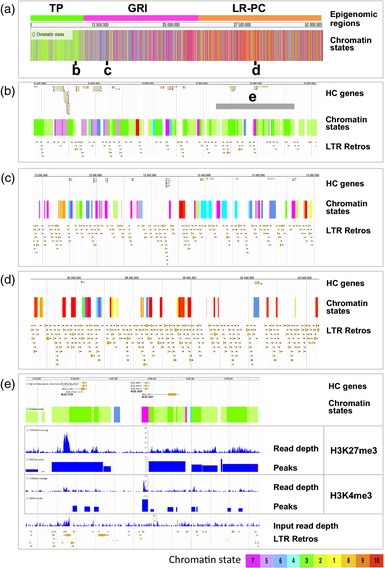
Fine structure of epigenomic features for barley chromosome 2H. (a) Locations of selected sub‐regions from the (b) telomere‐proximal (TP), (c) gene‐rich interior (GRI) and (d) low‐recombining pericentromeric (LR‐PC) regions of barley chromosome 2H are indicated on the chromatin state plot. Chromatin states are color‐coded as in Figures 3, 4, 5 (see key at bottom). (b) A 250‐kbp sub‐region of the TP region. The HC genes, chromatin states and annotated long terminal repeat (LTR) retrotransposons are also shown. The gray bar indicates a 75‐kbp genomic segment shown in greater detail in panel (e). (c) A 250‐kbp sub‐region of the GRI region. (d) A 250‐kbp sub‐region of the LR‐PC region. (e) Fine structure of the 75‐kb genomic segment from the TP region (see panel b). The chromatin immunoprecipitation next‐generation sequencing (ChIP‐seq) read depths and called peaks for H3K27me3 and H3K4me3 are shown, together with read depth for input DNA (negative control), HC genes, chromatin states and annotated LTR retrotransposons.

Figure 6 shows examples of local chromatin environments in 250‐kb segments of the three epigenomic regions identified above. Within the TP region (Figure 6b) gene density is high and chromatin states 2 and 3 predominate, with the former mostly intergenic and the latter mostly genic (Figure 6b, e). The active chromatin states 4–7 are also quite frequent, and these tend to map to known gene locations. Lastly, repressive states 1, 8, 9 and 10 are rare or absent and LTR retrotransposons are present at moderate levels. Closer inspection of a part of this region (Figure 6e, grey bar in Figure 6b) shows broad coverage by H3K27me3 of both genes and intergenic DNA. In the presence of H3K4me3 peaks this leads to state 3 and in its absence state 2 is found.

In the GRI region (Figure 6c) gene density is slightly lower than in the TP region, the H3K27me3‐containing states 2 and 3 are far less frequent, the active chromatin states 4–7 are very common, despite an increase in LTR retrotransposon density, and the four repressive states are present at low but increased levels. Lastly, areas devoid of modified histone peaks (state 11) are more common in the GRI region than the TP region. In the LR‐PC region (Figure 6d) genes are rare and LTR‐retrotransposon densities very high but active chromatin states are seen where genes are present. H3K27me3‐containing states are again rare in the LR‐PC and repressive states, particularly states 7–10, are much more common. Lastly, the area lacking modified histone peaks is even higher in the LR‐PC. We suggest that the latter is an artifact resulting from the extensive highly repetitious LTR retrotransposons in these regions which have a negative impact on read densities for the chromatin marks associated with them.

### Cytogenetic visualization of the barley epigenome

**Figure 7:**
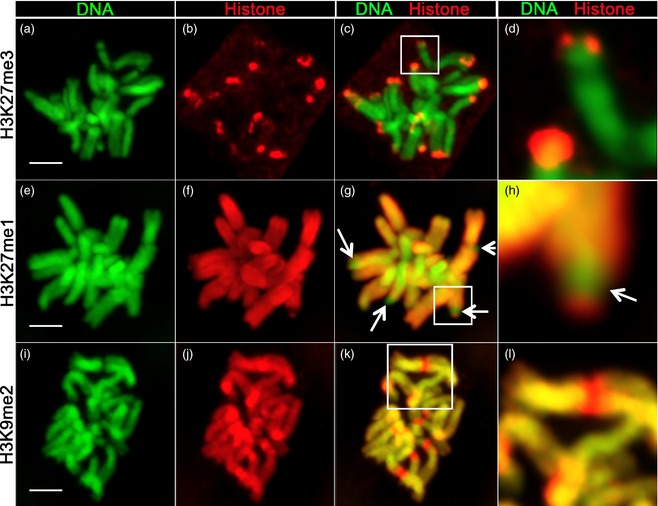
Immunolocalization of histone marks on barley mitotic chromosomes. All chromosome spreads are from mitotic barley root tip cells: top row, histone marks H3K27me3 (a–d); middle row, H3K27me1 (e–h); bottom row, H3K9me2 (i–l). For each histone modification, DNA (green, Hoechst stain), histone mark (red), merge of DNA/histone mark and close‐up of the boxed region are shown. Bars = 5 μm.

To confirm our ChIP‐seq‐based localizations for H3K27me3, H3K27me1 and H3K9me2 we used a cytogenetic approach to visualize these modified histones directly on barley mitotic chromosomes (Figure 7). Multiple TP regions are very strongly labelled with the H3K27me3 mark, with little signal visible elsewhere on the barley chromosomes (Figure 7a–d), consistent with our ChIP‐seq results (Figures 1 and 5). We checked the specificity of this H3K27me3 antibody batch (which was also used for the ChIP‐seq study) against a modified Histone Peptide Array. Our H3K27me3 antibody recognized H3K27me3‐containing peptides and displayed no cross‐reactivity against other histone modifications (Figure S4). We conclude that our ChIP‐seq experiments faithfully show the H3K27 genomic and cytogenetic distributions.

Our cytogenetic study also shows that H3K27me1 is broadly distributed across all barley chromosomes, in strong contrast to H3K27me3 (Figure 7e–h). Importantly, this repressive mark is also strongly depleted in multiple TP regions (Figure 7g, h, arrowed), mirroring the peak densities of this mark in Figures 1 and 5(j). H3K9me2 (Figure 7i–l) is also present across barley chromosomes but shows no cytogenetic evidence of depletion in the TP region. Rather, H3K9me2 is enriched in centromeric regions (Figure 7k, l), suggesting that the corresponding ChIP‐seq reads may not have mapped correctly to this highly repetitious region or it is not properly represented in our pseudogenome assembly. We conclude that: (i) the genomic distribution patterns deduced from the H3K27me1/3 ChIP‐seq data are supported by the cytogenetic data and underline an inverse distribution for H3K27me3 *vs*. H3K27me1; (ii) the cytogenetic H3K9me2 distribution does not support an inverse relationship for this mark.

## Discussion

### Modified histones and the distinction between active and inactive chromatin in barley

This study represents a comprehensive epigenetic description of the barley genome, which is the largest to have been assayed by ChIP‐seq to date. The genomic and genic distributions of the majority of modified histone marks reported here are broadly consistent with previous data from other plants (Li *et al*., 2008; Wang *et al*., 2009; He *et al*., 2010; Roudier *et al*., 2011). The well‐described gene‐associated modifications H3K4me2/3, H3K36me3 and H3K56ac show genomic profiles that follow genes (Figure 1) and the enrichment profiles for these four modifications across genes (Figure 2) support the conclusion that they are all involved in defining the genic epigenetic environment. Similarly, H3K9me2, which is a definitive heterochromatic mark in Arabidopsis (Lippman *et al*., 2004; Roudier *et al*., 2011), co‐localizes mainly with LTR retrotransposons and is predominantly intergenic (Figures 2, 3b, 4a, b and 6). We conclude that H3K9me2 performs the same role as described for Arabidopsis of defining the repressed constitutive heterochromatic state (Lippman *et al*., 2004; Roudier *et al*., 2011; Sequeira‐Mendes *et al*., 2014).

### Chromatin state analysis reveals an unexpected higher‐order structure of barley chromosomes involving H3K27me1 and H3K27me3

Our chromatin state analysis has revealed a chromosome‐scale epigenomic higher‐order structure for barley chromosome arms, with gene‐rich TP regions that are heavily covered by genic and intergenic H3K27me3 and depleted for H3K27me1, juxtaposed with GRI regions that are rich in active chromatin states and greatly depleted for H3K27me3. In rice the chromosomal distribution of H3K27me3 is indistinguishable from that of genes (He *et al*., 2010), and in maize it shows a similar profile to mRNA across chromosomes (Gent *et al*., 2012). In rye mitotic and barley meiotic cells high levels of H3K27me3 are visible near telomeres by cytogenetics (Carchilan *et al*., 2007; Higgins *et al*., 2012) but these studies cannot distinguish genic from intergenic H3K27me3.

Roughly 30–40% of barley genes reside in each of the TP and GRI regions (Baker *et al*., 2014), which appear to employ different mechanisms for global epigenomic regulation. In the TP region H3K9me2‐mediated constitutive heterochromatin is rare, presumably because LTR retrotransposons are also rare here (Figures 5k, l and 6b). In its place H3K27me3 is used to maintain a widespread facultative heterochromatic state (Figure 6b, e). In animals H3K27me3 mediates the establishment of facultative heterochromatin via deposition at polycomb response elements by the Polycomb Repressor Complex 2 followed by spreading to adjacent regions. H3K27me3 does not extend over large genomic regions in Arabidopsis (Turck *et al*., 2007) or rice (Wu *et al*., 2011) but it clearly does in the barley TP region, where the majority of this histone mark is found. State 2 is the dominant source of H3K27me3 in the barley genome, accounting for 83 Mbp compared with 29 Mbp for state 3 and the large majority of this state is intergenic.

How is the heterochromatic state controlled in the GRI and LR‐PC regions? Both H3K9me2 and H3K27me1 are present in these regions, both are characteristic marks for constitutive heterochromatin in Arabidopsis (Mathieu *et al*., 2005; Jacob *et al*., 2010) and H3K27me1 acts similarly in animals (Peters *et al*., 2003). We therefore suggest that the constitutive heterochromatic state is specified by H3K27me1 in the GRI and LR‐PC regions. The inverse distribution of H3K27me1 and H3K27me3 (Figure 5d, j) and their defining roles for the TP and GRI regions lead us to speculate that the methylation status of H3K27 is central to the regional epigenomic structure reported here. Furthermore, the tight inverse relationship between H3K27me3 and H3K27me1 implies a mutually repressive interaction between these two marks but further experiments are clearly needed to test this hypothesis.

Fascinatingly, a similar situation may operate in *Neurospora*, where H3K27me3 also shows biased abundance towards sub‐telomeric regions and against the heterochromatic H3K9me3 mark (Jamieson *et al*., 2013). It is tempting to speculate that this shared property points to a mechanistic connection, and we do observe inverse distribution between states containing H3K27me3 and [H3K9me2+ H3K27me1] (Figure S5; *R* ^2^ = 0.43, *P *<* *0.0001). However, the huge evolutionary distance between plant and fungal taxa and the prevalence of broadly distributed H3K27me3 in most lineages of the animal and plant kingdoms suggests to us that this is a case of convergent evolution rather than a common property inherited from a common ancestor.

How stable are TP regions on an evolutionary scale? A possible clue is provided by chromosomes 4HL and 5HL. The former has a very weak TP region and the latter shows a complex structure, with interspersed TP and GRI regions (Figure S3). The marked region on 5H in Figure S3 has been translocated to its current location from chromosome 4HL since the divergence of *Lolium* and the Triticeae, more than 10 million years ago (King *et al*., 2013; Baker *et al*., 2014). We suggest that the ancient 5H TP region has survived this translocation, the translocated 4H segment (marked by a box in Figure S3) has also retained its TP region and the truncated chromosome 4H has not rebuilt a strong TP region since the translocation event.

### Histone modification, chromatin state and barley gene expression

Our exploration of the relationship between chromatin state and divergence of gene expression between tissues has revealed interesting results concerning the biological functions of several barley chromatin states. First, the two major states that are associated with low DGE levels, namely states 5 and 6, carry strong inputs from H3K36me3, and these are the major states involved with this modification (Figures 3 and 4c). We therefore suggest that H3K36me3 plays a role in constitutive gene expression in barley. Interestingly, these two H3K36me3‐bearing states are also associated with the highest gene expression levels in the barley seedling (Figure S6). These collective data are consistent with the role of H3K36me3 in Arabidopsis acting antagonistically to H3K27me3 in controlling *FLC* gene expression (Yang *et al*., 2014), in maize of enrichment in genes expressed at the same level between root and shoot tissue (Wang *et al*., 2009), and in Drosophila of being enriched in housekeeping genes (Filion *et al*., 2010; Brown and Bachtrog, 2014).

Second, the heavy bias in enrichment of chromatin state 3 towards high DGE levels in the barley seedling (Figure 4c) suggests strongly that H3K27me3 acts in barley to repress gene expression in tissue subsets. Furthermore, averaged total gene expression levels for the genic H3K27me3‐bearing state 3 are lower than any other genic state (Figure S6). Lastly, plots of averaged DGE levels across the barley genome show increases towards the telomeres (Figure S7). We conclude that H3K27me3‐mediated gene repression is a major facilitator of DGE in the barley seedling. However, this cannot be the only way to control barley DGE because H3K27me3 is predominantly found only in the TP region.

### Roles for other histone modifications in barley

The other two histone modifications studied here, H3K27me2 and H3K9me3, have also yielded unexpected results. These two modifications share a similar genic and genomic distribution profile (Figure 1). Peak sharing supports an affinity between these two Class III modifications (Figure 3a), yet peaks for both H3K9me3 and H3K27me2 are rare in barley genes (Table S2), suggesting a predominantly intergenic location. In Arabidopsis H3K9me3 is an exclusively genic mark and H3K27me2 resides in both the heterochromatin and the euchromatin, where it has strong overlap with H3K27me3, which is also strongly genic (Turck *et al*., 2007; Roudier *et al*., 2011). However, in rice H3K9me3 has been implicated in repression of the Tos17 LTR retrotransposon (Qin *et al*., 2010) and in rye it is found in sparse punctate regions, consistent with our genomic data (Carchilan *et al*., 2007). To our knowledge the only previous reports for the location of H3K27me2 in cereals are cytogenetic (Shi and Dawe, 2006; Carchilan *et al*., 2007; Jin *et al*., 2008). These reports disagree on the genomic location, with a reported heterochromatic location in maize and a euchromatic location in rye. These results are difficult to reconcile, and it is possible that the specificities of the antibodies used are responsible. Our H3K27me2 data are consistent with the maize data.

## Conclusions

This study has described the detailed structure of the barley epigenome for the first time and reveals an unexpected higher‐order chromatin structure comprising three epigenomic regions defined primarily by the relative abundances of H3K27me3, H3K27me1 and a group of active chromatin marks. We expect that this organization will be common to the other Triticeae cereals, including wheat. The regional role of H3K27me3 in specifying genic and intergenic repressive functions in the TP regions is highly reminiscent of its role in animals and fungi in determining the facultative heterochromatic state. We propose that the inverse relationship between the distributions of H3K27me3 and H3K27me1, which delineates the boundary between the TP and GRI regions, is an important component in higher‐order organization of the barley epigenome.

Our study is to some extent just the beginning of the description of the chromatin landscape of the barley genome. We have only addressed one mixed tissue, namely the developing seedling, and different tissues will certainly yield different chromatin profiles (Makarevitch *et al*., 2013). Likewise, we have only studied nine of the more than 50 known histone modifications, and we have only just begun to study the detailed properties of the genic states described here with regard to the differing types of genes containing them. Nevertheless, the findings reported here and the underpinning data available via our genome browser provide a coherent description of the barley epigenome, with new findings that both raise interesting questions and provide a framework to address them in the future.

## Experimental Procedures

### Plant materials

Seeds of *Hordeum vulgare* cv. Morex were germinated on water‐soaked filter paper in Petri dishes and grown at room temperature 20°C until the leaf tissues were about 10 cm long (about 10 days). Plant material from 18 whole seedlings (roughly 3 g) was harvested and divided into three replicates. For cytology, seeds were germinated on wet filter paper for 5 days before harvesting the root tips.

### The ChIP‐seq analysis

Pooled barley seedlings were cross‐linked under vacuum in 1% (w/v) formaldehyde for 15 min at room temperature. The cross‐linking reaction was quenched by the addition of 0.125 m glycine and the vacuum was reapplied for a further 5 min. The cross‐linked plant material was flash‐frozen in liquid nitrogen and ground to a fine powder. For all the following steps the Abcam EpiSeeker Plant kit (catalog no. ab117137; http://www.abcam.com/) was used according to the manufacturer's instructions. Nuclei were extracted and chromatin was sonicated using a Diagenode Biorupter Plus (http://www.diagenode.com/) with 10 cycles at 4°C at high power of (30 sec pulse/60 sec cooling). The resulting sheared chromatin was pooled from the three replicates and 100‐μl aliquots were immunoprecipitated in triplicate each with 3 μl of 10 antibodies (Table S4) for 90 min. Three control input chromatin aliquots (5 μl) were taken prior to immunoprecipitation (‘input DNA’) and subsequently treated in the same way as the immunoprecipitated samples, apart from the immunoprecipitation step. Reverse cross‐linking was performed for all samples and DNAs were extracted and purified using columns from the EpiSeeker kit. Illumina libraries were constructed from the resulting DNA fractions using a Diagenode MicroPlex kit (catalog no. C05010010) according to the manufacturer's instructions. The barcoded DNA libraries were pooled with 8× multiplexing per lane and sequenced using the Illumina HiSeq 2000 (101‐bp paired end reads; https://www.illumina.com/).

### Validation of H3K27me3 antibody specificity

The H3K27me3 antibody batch used in this study was checked using the Active Motif MODified Histone Peptide Array (catalog no. 13005; http://www.activemotif.com/) using the manufacturer's protocol.

### Cytology and microscopy

Root tips were incubated in cold water for 6 h and fixed in 4% formaldehyde for 30 min. Root tips were digested in a mixture of 1% Cellulase (ONOZUKA R10) and 1% Pectolyase Y23 (Duchefa, https://www.duchefa-biochemie.com/) in 0.01 m citrate buffer for 30 min at 37°C (Higgins *et al*., 2012). Roots tips were rinsed twice in 1× PBS/0.5% Triton X‐100 and three to four tips were squashed in between two polylysine slides and air dried before applying 30 μl of primary antibody solution consisting of individual rabbit histone antibody (Table S4) diluted (1:100) in 1× PBS/0.5% Triton X‐100. Slides were incubated for 2 days at 4°C, rinsed and incubated for an extra 2 h at room temperature in the secondary antibody solution consisting of anti‐rabbit Alexa Fluor^®^488. Slides were washed, counterstained with Hoechst 33342 (2 μg ml^−1^; Life Technologies, http://www.thermofisher.com/) and mounted in Vectashield^®^ (H‐1000; Vector Laboratories, http://vectorlabs.com/uk/). Three‐dimensional confocal stack images were acquired with a LSM‐Zeiss 710 instrument using laser light (405, 488, 561 and or 594 nm; http://www.zeiss.com/) sequentially. Images were processed with fiji (Schindelin *et al*., 2012) and imaris 7.7.2 (Bitplane, http://www.bitplane.com/) for extra rendering.

### Mapping ChIP‐seq reads to the barley genome and peak calling

The ChIP‐seq reads were mapped to a manually assembled Morex genome based on the Morex v.3 genome assembly that is anchored to 6474 distinct centimorgan (cM) bins of a high‐density Morex × Barke genetic map and contains associated TE annotation (IBGSC 2012, Mascher *et al*., 2013). Contigs with known cM map locations were ordered on chromosomes then randomly ordered within genetic map bins and finally assembled into a FASTA file, with a string of NNNNNNNNNN between each contig. Overall, 766 611 contigs at an overall density of 616 contigs Mb^−1^ were included in the manually assembled genome. Reads were mapped using star v.2.3.0.1 (Dobin *et al*., 2013) with parameters set to perform adaptor trimming, and with additional parameters to limit the minimum mapped paired end read length to 72 bp (–outFilterScoreMinOverLread 0.36–outFilterMatchNminOverLread 0.36), to limit the mismatch rate to 5% (–outFilterMismatchNoverLmax 0.05) and to disable mapping across splice junctions (–alignIntronMin 2–alignIntronMax 1). Non‐uniquely‐mapping reads were limited to 10 potential mappings; star marks nine as secondary alignments and the remaining mapping is either the highest scoring or randomly selected from alignments of equal quality. Secondary mappings were removed with SAmtools v.0.1.18 view (Li *et al*., 2009) then PCR duplicates were removed with picard‐tools v.1.51 MarkDuplicates (http://broadinstitute.github.io/picard/).

Three different peak calling software programs, ccat, findpeaks and sissrs (Fejes *et al*., 2008; Jothi *et al*., 2008; Xu *et al*., 2010), were trialled, using default parameters. Quantitative PCR of eight genes (using H3K56ac data) was used to compare software performance and validate the ChIP‐seq peak data (Table S5). Quantitative PCR was performed using Maxima SYBR Green/ROX qPCR Master Mix (catalog no. K0222; Thermo Scientific, http://www.thermoscientific.com/) with the H3K56ac DNA Illumina libraries as templates. The primers used are listed in Table S5. The qPCR program was: 95°C for 15 min; 40 cycles of 95°C for 15 sec, 60°C for 30 sec, 72°C for 30 sec. Peaks were called from the qPCR data if the fold change (2^[*C*(*t*) Input – *C*(*t*) Sample]^) was greater than 5. Additionally, if no qPCR product was detected in the input but was in the sample, a peak was called and if no qPCR product was detected in the sample, a peak was not called.

Following the comparison of software performance (Table S5), ccat (Xu *et al*., 2010) was chosen and used for this study. The default histone peak calling parameters (with three alterations of fragmentSize = 100, slidingWinSize = 1000, minScore = 2.0) were used for calling H3, H3K4me2, H3K9me3, H3K9me3, H3K27me1, H3K27me2, H3K27me3 and H3K36me3 peaks. Default transcription factor peak calling parameters (with four alterations of fragmentSize = 100, minCount = 4, minScore = 2.0, isStrandSensitiveMode = 0) were used for calling H3K4me3 and H3K56ac peaks, which are sharper.

Peaks were called against the input DNA samples as a background. ccat calculates fold change as the proportion of sample reads over the background reads, with the number of reads normalized by library size (Xu *et al*., 2010). Data sets were then filtered to retain only peaks with a fold change greater than four. The top 100 000 peaks for each replicate were retained. Peaks were retained for study only if they were observed in at least two independent replicates, using BEDtools v.2.20.0 merge and intersect (Quinlan and Hall, 2010). diffbind v.1.12.0 (Stark and Brown, 2011) was used to perform correlation analyses of histone modifications.

### Assigning peaks to genes and analysis of histone modifications in different gene expression environments

The RNA‐seq data from the IBGSC (2012) project were used to divide gene transcripts into zero‐, low‐, mid‐ and high‐expression groups. Mean reads per kilobase per million (RPKM) of seedling root and seedling leaf tissue was calculated and used as the representative level of gene expression in the seedling tissue. Of the 21 965 genes (58 819 transcripts) with known physical location and gene expression, 774 genes (980 transcripts) had zero gene expression. The remaining genes were split evenly into low‐, mid‐ and high‐expression groups, corresponding to 7074 high‐ and low‐expression genes and 7063 mid‐expression genes. The total numbers of low‐expression transcripts were 17 882, 21 852 and 18 105, respectively, for low‐, mid‐ and high‐expression transcripts.

The software package genomictools (Tsirigos *et al*., 2012) was used to relate peaks to gene transcripts using the program genomic overlaps offset. Peaks were required to be within 1.5 kb of the TSS or TES in order to be attributable to the transcript itself. Peaks assigned to gene transcripts were then separated based on expression level. Custom Java code was used to generate histogram data across 100 equally sized bins along transcripts and an additional 100 bins from the TSS to 1 kb upstream of the TSS and from the TES to 1 kb downstream of the TES. The mean fold change in each bin was calculated, representing the genic profile of each histone modification, and the output data were plotted in R.

To utilize the fold change information from large peaks extending further than 1.5 kb upstream or downstream the mean fold change was also calculated by intersecting peaks with genes and assigning an overall fold change for each intersected gene based on the fold change for the peak.

### Deriving chromatin states for the barley epigenome

Chromatin states were learned considering models from two to twenty states by applying the chromHMM v.1.10 hidden Markov model algorithm with a bin size of 150 bp (Ernst and Kellis, 2012). chromHMM comparemodels was used to compare all the models with each other. One‐ to 19‐state models were explored and an 11‐state model (10 states with histone modifications, 1 state without any modifications) was chosen after inspecting the relative performances of the models versus a 20‐state model (Figure S2). The 11‐state model has the lowest number of states that capture all chromatin state information from the 20‐state model with a correlation of >0.7 for emission parameters versus the 20‐state model whilst retaining biologically meaningful state assignments.

### Relating chromatin states to barley genomic, genic and gene expression parameters

chromHMM overlapenrichment (Ernst and Kellis, 2012) was used to determine enrichments for chromatin states relative to both genomic and genic annotations (Figure 4a, b) and to gene bins derived from DGE data (Figure 4c). Average gene expression values in each chromatin state were determined from RPKM values from seedling root and leaf RNA‐seq data (IBGSC 2012) and BEDtools v.2.20.0 intersect (Quinlan and Hall, 2010) was used to assign genes to states; gene expression values for each state were plotted as boxplots using R.

Differential gene expression was estimated for genes by comparing root and leaf RNA‐seq data for the same stage of the barley seedling as used for the ChIP‐seq analysis (IBGSC 2012). Genes were ranked by root expression level and a rank value was obtained for each gene by dividing its rank position by the total number of genes (Yu *et al*., 2010). This method was repeated for leaf gene expression. The DGE level for each gene was then expressed as the difference between its two rank values, between zero (zero DGE) and one (high DGE). Genes were binned into five equally sized DGE bins at intervals of 0.2. Enrichment in chromatin states within DGE bins was calculated using chromHMM overlapenrichment (Ernst and Kellis, 2012). Rolling averages for DGE across pseudochromosomes (Figure S7) were calculated in two passes with window sizes of 25 genes and then again with 250 genes with the R package TTR.

### A genome browser for the barley epigenome

All finished data from this study was imported into a JBrowse genome browser (Skinner *et al*., 2009) set up at http://ics.hutton.ac.uk/jbrowse/barley-chip. The ChIP‐seq peak data, together with corresponding contig, gene and TE annotations for our genome assembly, can be downloaded at https://ics.hutton.ac.uk/barley-epigenome/.

## Accession Numbers

All sequence data from this article can be found in the EMBL/GenBank Short Read Archive under accession number PRJEB8068 (http://www.ebi.ac.uk/ena/data/view/PRJEB8068).

## Supporting information


**Figure S1.** Peak densities of histone modifications across barley chromosomes.

**Figure S2.** Performance characteristics of different chromatin state models.

**Figure S3.** High‐order epigenomic structures of barley chromosomes.

**Figure S4.** H3K27me3 histone antibody validation.

**Figure S5.** Relationship between distribution of H3K27me3 and H3K27me1/H3K9me2.

**Figure S6.** Average gene expression levels in chromatin states.

**Figure S7.** Differential gene expression in the barley genome.

**Table S1.** Peak numbers for histone modifications in this study.

**Table S2.** Peak numbers for modified histones that are associated with genes.

**Table S3.** Emissions for chromatin state models.

**Table S4.** Antibodies used in this study.

**Table S5.** Quantitative PCR validation of peak finding software.
