# Supplementary material for: Chromatin state analysis of the barley epigenome reveals a higher‐order structure defined by H3K27me1 and H3K27me3 abundance
Source: Plant J. 2015 Sep 9;84(1):111–24. doi: 10.1111/tpj.12963 (PMC4973852; doi:10.1111/tpj.12963)
Supplement: Supplementary file 1 — Figure S1. Peak densities of histone modifications across barley chromosomes. [file TPJ-84-111-s001.pdf]

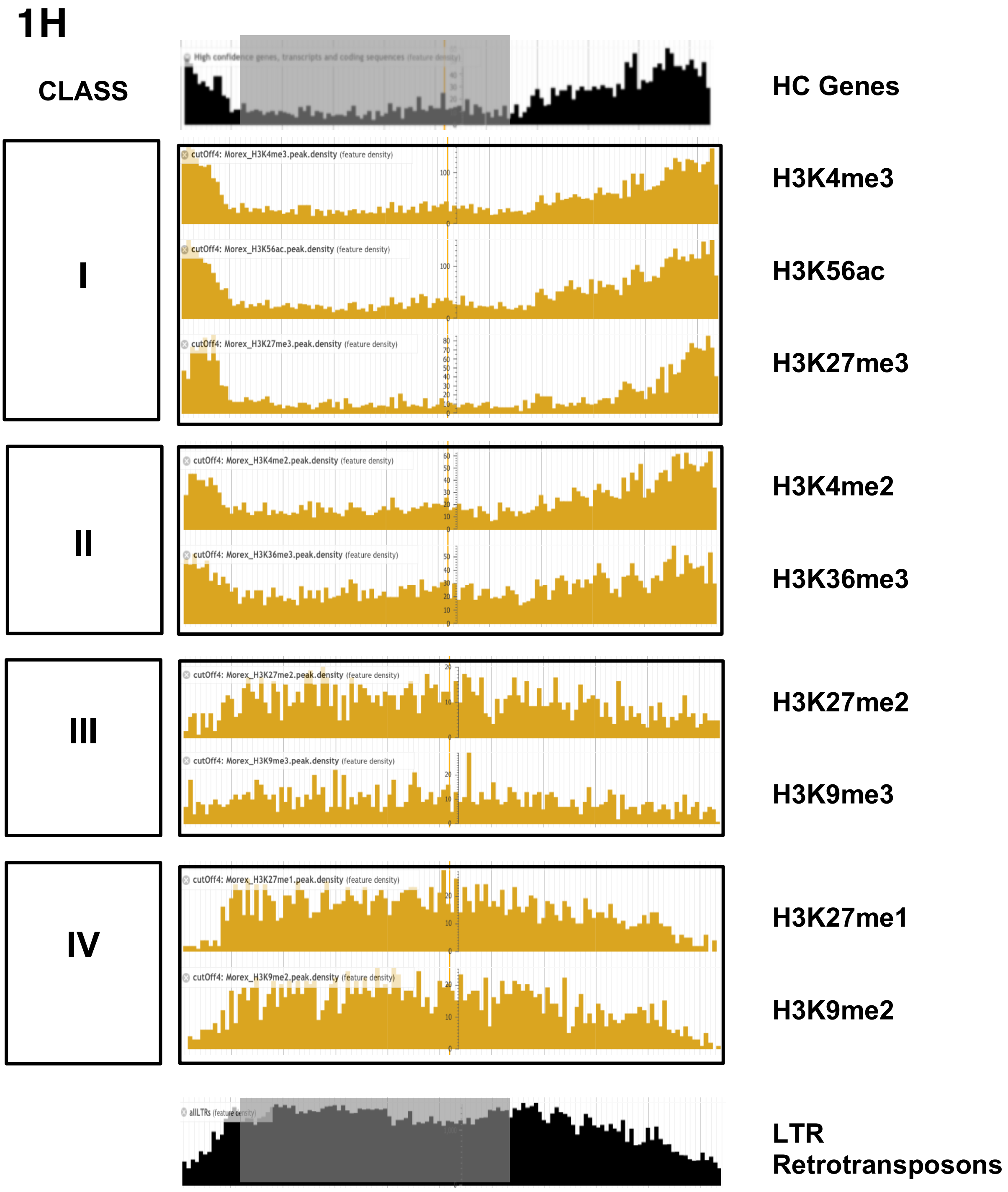

1H
CLASS
I
II
III
IV
HC Genes
H3K4me3
H3K56ac
H3K27me3
H3K4me2
H3K36me3
H3K27me2
H3K9me3
H3K27me1
H3K9me2
LTR Retrotransposons
cutOff4: Morex_H3K4me3.peak.density (feature density)
cutOff4: Morex_H3K56ac.peak.density (feature density)
cutOff4: Morex_H3K27me3.peak.density (feature density)
cutOff4: Morex_H3K4me2.peak.density (feature density)
cutOff4: Morex_H3K36me3.peak.density (feature density)
cutOff4: Morex_H3K27me2.peak.density (feature density)
cutOff4: Morex_H3K9me3.peak.density (feature density)
cutOff4: Morex_H3K27me1.peak.density (feature density)
cutOff4: Morex_H3K9me2.peak.density (feature density)

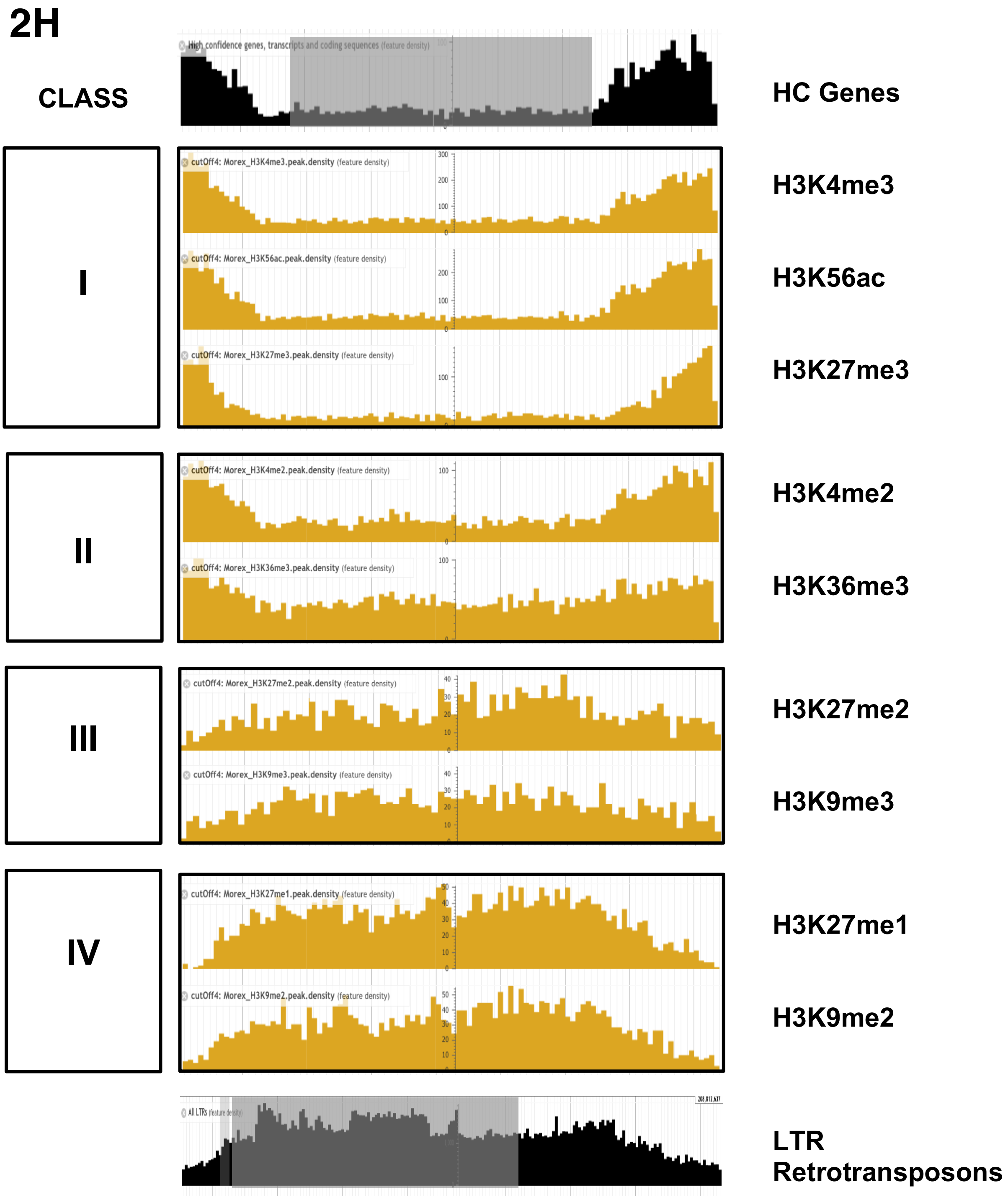

2H
CLASS
I
II
III
IV
HC Genes
H3K4me3
H3K56ac
H3K27me3
H3K4me2
H3K36me3
H3K27me2
H3K9me3
H3K27me1
H3K9me2
LTR
Retrotransposons

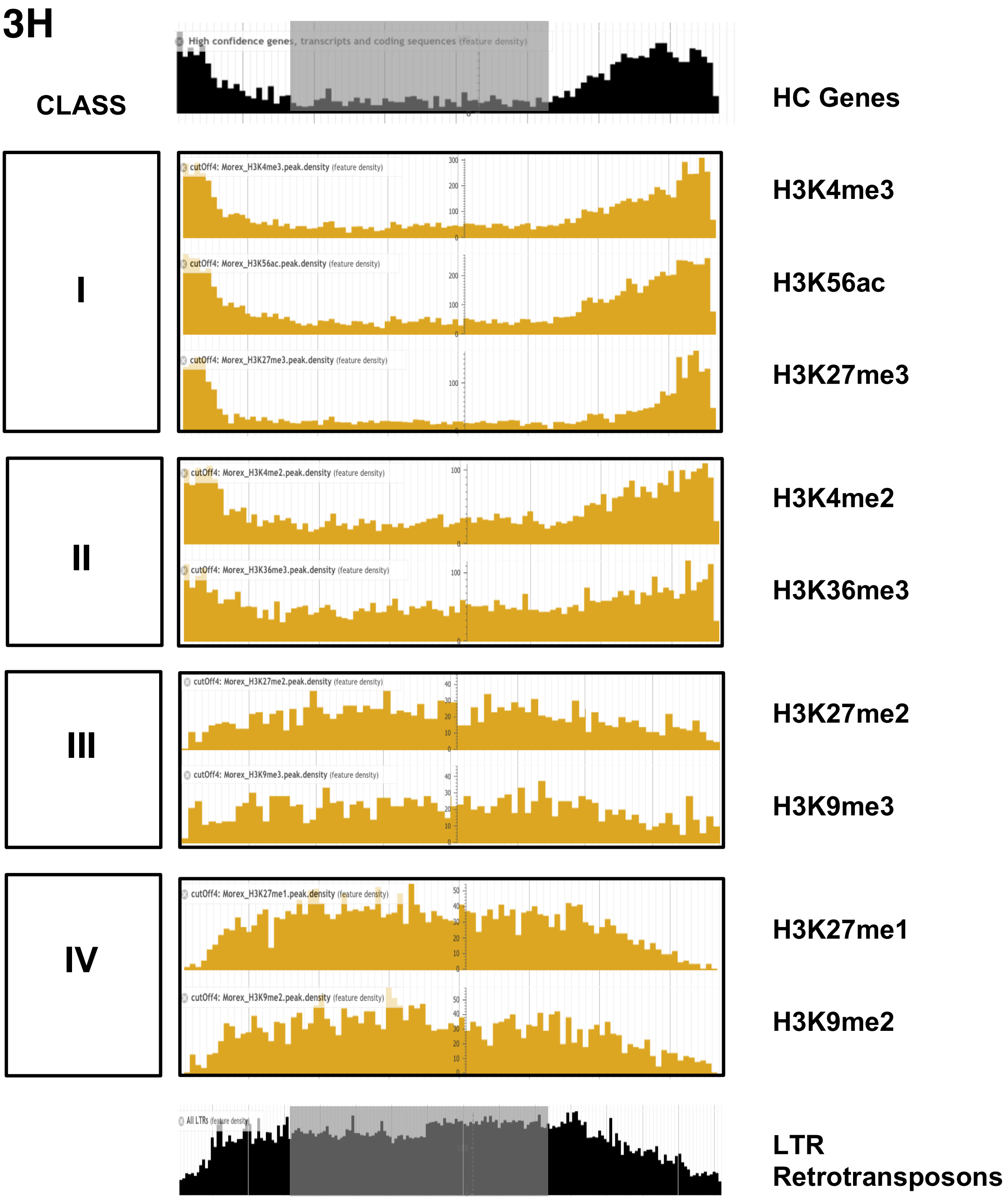
3H
CLASS
I
II
III
IV
High confidence genes, transcripts and coding sequences (feature density)
cutOff4: Morex_H3K4me3.peak.density (feature density)
cutOff4: Morex_H3K56ac.peak.density (feature density)
cutOff4: Morex_H3K27me3.peak.density (feature density)
cutOff4: Morex_H3K4me2.peak.density (feature density)
cutOff4: Morex_H3K36me3.peak.density (feature density)
cutOff4: Morex_H3K27me2.peak.density (feature density)
cutOff4: Morex_H3K9me3.peak.density (feature density)
cutOff4: Morex_H3K27me1.peak.density (feature density)
cutOff4: Morex_H3K9me2.peak.density (feature density)
All LTRs (feature density)
HC Genes
H3K4me3
H3K56ac
H3K27me3
H3K4me2
H3K36me3
H3K27me2
H3K9me3
H3K27me1
H3K9me2
LTR
Retrotransposons

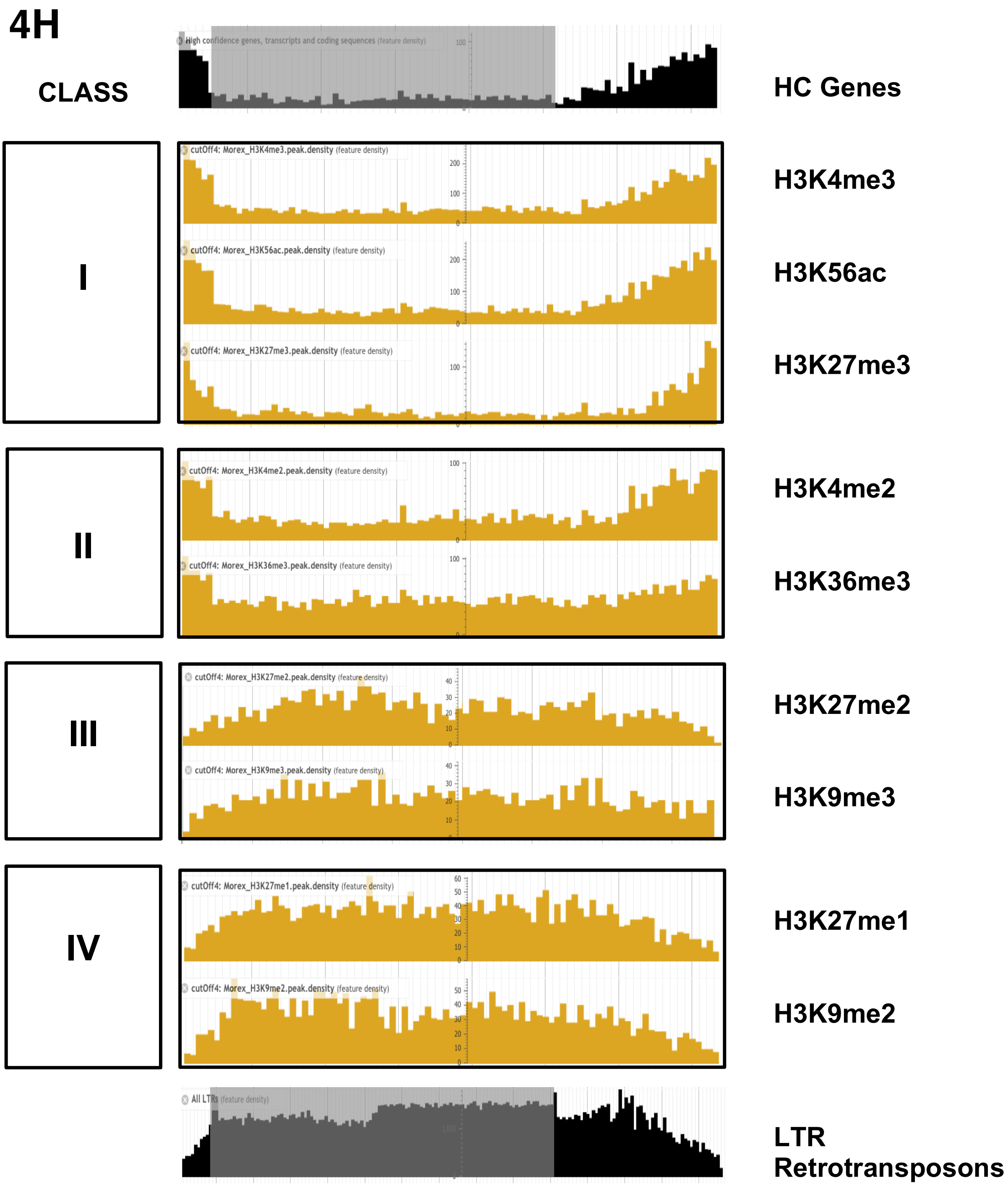

4H
CLASS
HC Genes
High confidence genes, transcripts and coding sequences (feature density)
I
cutOff4: Morex_H3K4me3.peak.density (feature density)
H3K4me3
cutOff4: Morex_H3K56ac.peak.density (feature density)
H3K56ac
cutOff4: Morex_H3K27me3.peak.density (feature density)
H3K27me3
II
cutOff4: Morex_H3K4me2.peak.density (feature density)
H3K4me2
cutOff4: Morex_H3K36me3.peak.density (feature density)
H3K36me3
III
cutOff4: Morex_H3K27me2.peak.density (feature density)
H3K27me2
cutOff4: Morex_H3K9me3.peak.density (feature density)
H3K9me3
IV
cutOff4: Morex_H3K27me1.peak.density (feature density)
H3K27me1
cutOff4: Morex_H3K9me2.peak.density (feature density)
H3K9me2
All LTRs (feature density)
LTR
Retrotransposons

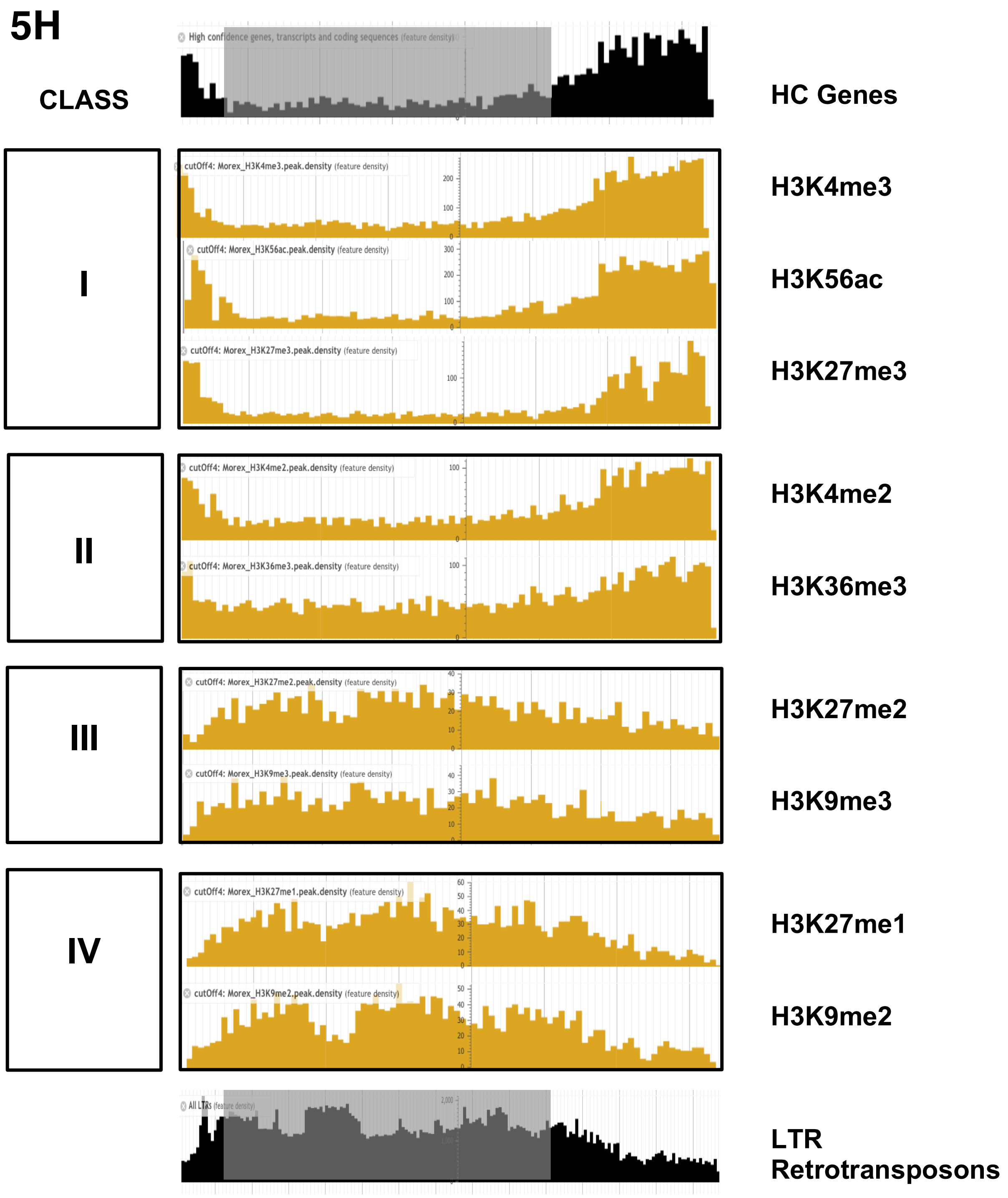
5H
CLASS
High confidence genes, transcripts and coding sequences (feature density)
HC Genes
I
cutOff4: Morex_H3K4me3.peak.density (feature density)
H3K4me3
cutOff4: Morex_H3K56ac.peak.density (feature density)
H3K56ac
cutOff4: Morex_H3K27me3.peak.density (feature density)
H3K27me3
II
cutOff4: Morex_H3K4me2.peak.density (feature density)
H3K4me2
cutOff4: Morex_H3K36me3.peak.density (feature density)
H3K36me3
III
cutOff4: Morex_H3K27me2.peak.density (feature density)
H3K27me2
cutOff4: Morex_H3K9me3.peak.density (feature density)
H3K9me3
IV
cutOff4: Morex_H3K27me1.peak.density (feature density)
H3K27me1
cutOff4: Morex_H3K9me2.peak.density (feature density)
H3K9me2
All LTRs (feature density)
LTR Retrotransposons

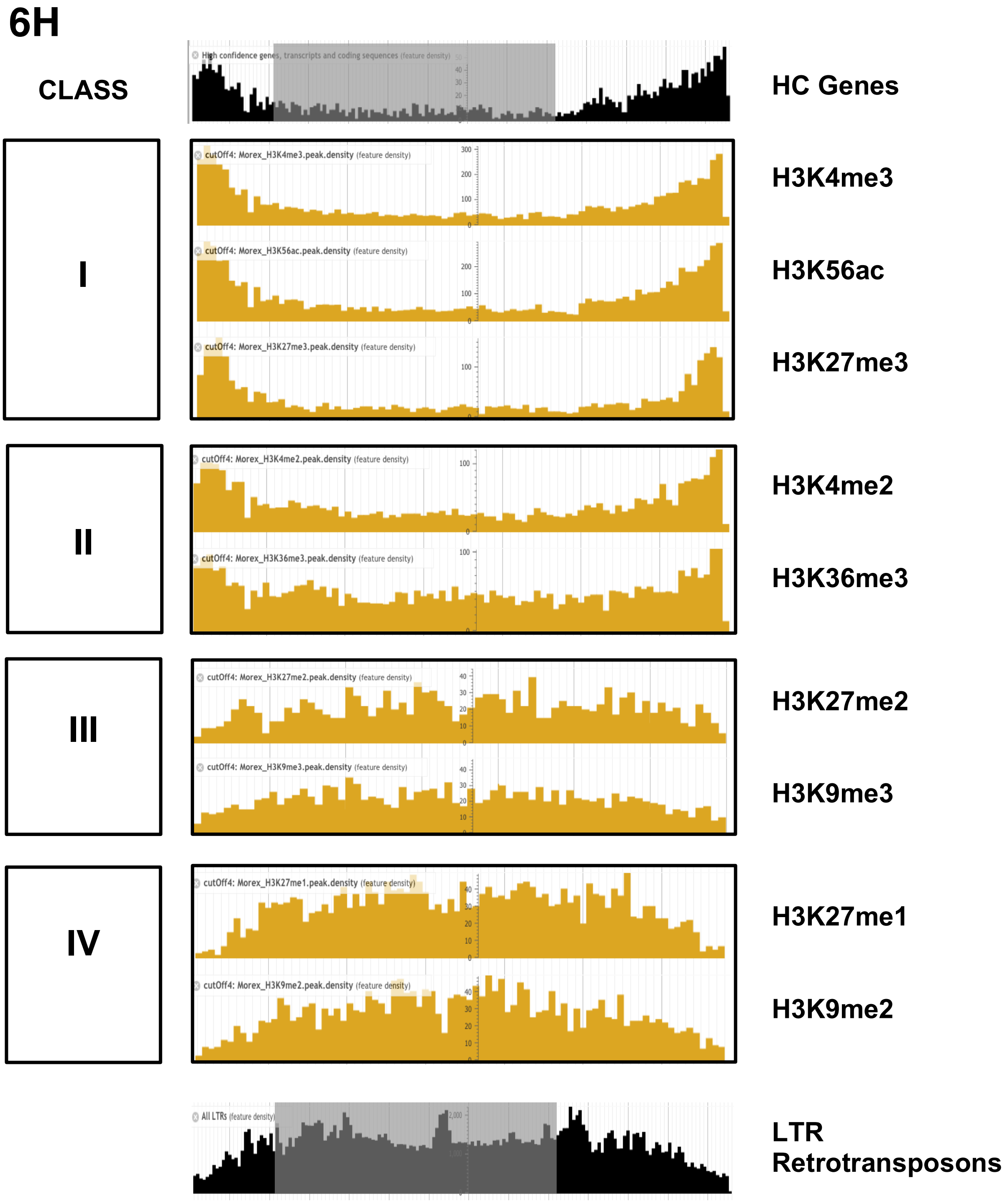
6H
CLASS
I
II
III
IV
HC Genes
H3K4me3
H3K56ac
H3K27me3
H3K4me2
H3K36me3
H3K27me2
H3K9me3
H3K27me1
H3K9me2
LTR Retrotransposons
High confidence genes, transcripts and coding sequences (feature density)
cutOff4: Morex_H3K4me3.peak.density (feature density)
cutOff4: Morex_H3K56ac.peak.density (feature density)
cutOff4: Morex_H3K27me3.peak.density (feature density)
cutOff4: Morex_H3K4me2.peak.density (feature density)
cutOff4: Morex_H3K36me3.peak.density (feature density)
cutOff4: Morex_H3K27me2.peak.density (feature density)
cutOff4: Morex_H3K9me3.peak.density (feature density)
cutOff4: Morex_H3K27me1.peak.density (feature density)
cutOff4: Morex_H3K9me2.peak.density (feature density)
All LTRs (feature density)

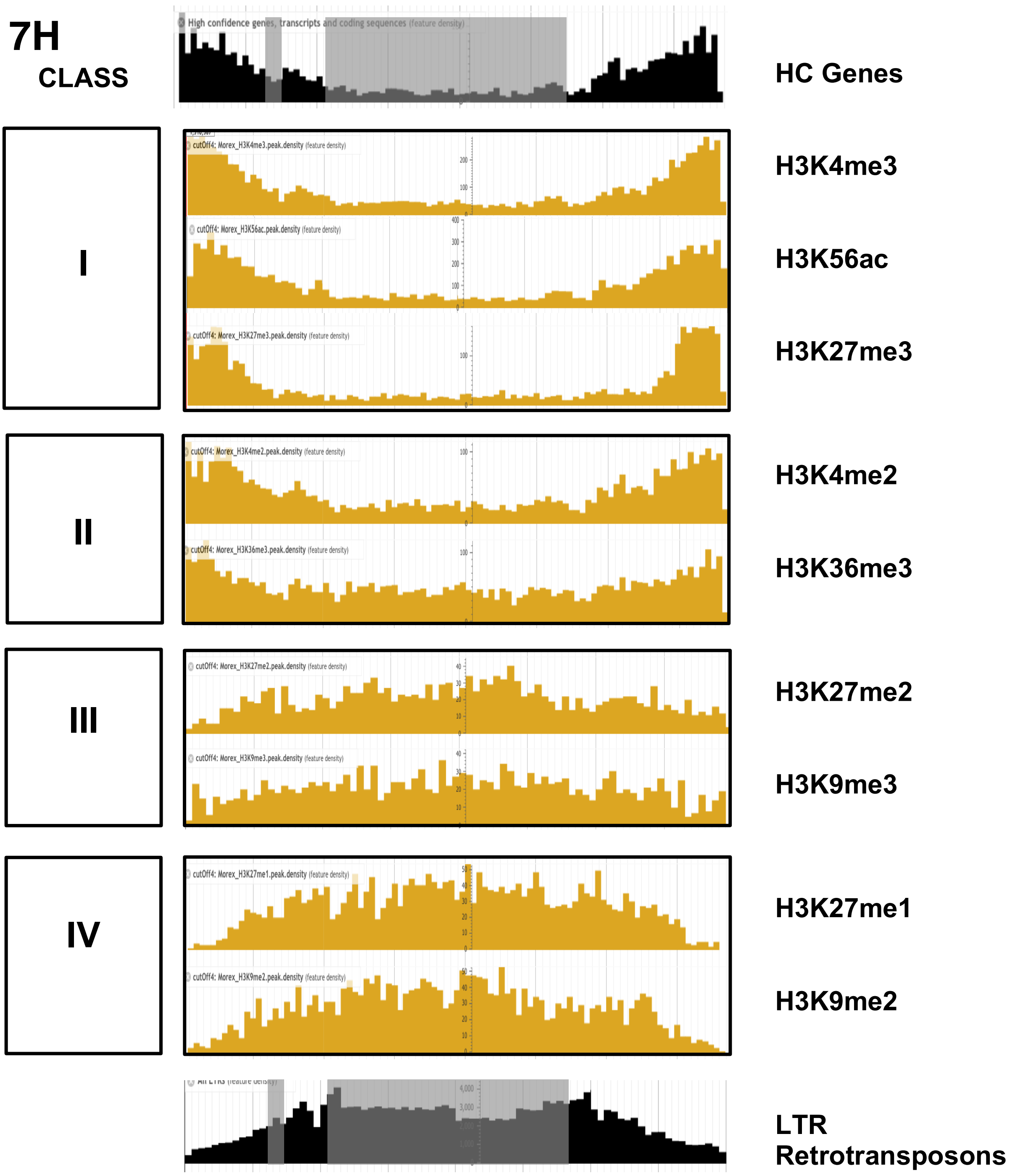

Figure S1: Peak densities of histone modifications across barley chromosomes: Local peak densities for the nine histone modifications studied here are plotted against pseudo-physical positions on barley chromosomes in JBrowse (see Methods). Distribution Classes I-IV are described in the text. The distributions of HC genes and LTR retrotransposons are also shown and the location of the LR-PC region (Baker *et al.* 2014) is indicated in grey.
