# Supplementary material for: Chromatin state analysis of the barley epigenome reveals a higher‐order structure defined by H3K27me1 and H3K27me3 abundance
Source: Plant J. 2015 Sep 9;84(1):111–24. doi: 10.1111/tpj.12963 (PMC4973852; doi:10.1111/tpj.12963)
Supplement: Supplementary file 2 — Figure S2. Performance characteristics of different chromatin state models. [file TPJ-84-111-s002.pdf]

	1	2	3	4	5	6	7	8	9	10	11	12	13	14	15	16	17	18	19	20
**1**	0.482	0.520	0.759	0.715	0.724	0.724	0.728	0.729	0.845	0.812	0.812	0.812	0.812	0.812	1.000	1.000	1.000	1.000	1.000	1.000
**2**	0.661	0.694	0.968	0.830	0.852	0.852	0.861	0.865	0.867	0.922	0.921	0.921	0.921	1.000	1.000	1.000	1.000	1.000	1.000	1.000
**3**	0.546	0.546	0.785	0.848	0.851	0.851	0.852	0.852	0.853	0.931	0.934	0.934	0.934	1.000	1.000	1.000	1.000	1.000	1.000	1.000
**4**	0.386	0.381	0.601	0.877	0.858	0.858	0.849	0.845	0.842	0.953	0.953	0.953	0.953	0.953	1.000	1.000	1.000	1.000	1.000	1.000
**5**	0.701	0.674	0.660	0.660	0.659	0.659	0.658	0.658	0.657	0.644	0.706	0.706	0.711	0.711	0.711	0.966	0.966	0.991	0.999	1.000
**6**	0.477	0.443	0.963	0.963	0.961	0.961	0.960	0.955	0.952	0.959	0.993	0.993	0.994	0.994	0.994	0.997	0.997	1.000	1.000	1.000
**7**	0.465	0.471	0.692	0.692	0.693	0.693	0.693	0.693	0.696	0.700	0.721	0.721	0.991	0.991	0.991	0.995	0.995	1.000	1.000	1.000
**8**	0.207	0.248	0.365	0.659	0.659	0.807	0.807	0.804	0.803	0.803	0.803	0.985	0.985	0.985	0.985	0.985	1.000	1.000	1.000	1.000
**9**	0.382	0.437	0.581	0.947	0.947	0.971	0.972	0.973	0.973	0.973	0.973	0.931	0.931	0.931	0.931	0.931	1.000	1.000	1.000	1.000
**10**	0.189	0.220	0.265	0.424	0.425	0.443	0.443	0.445	0.646	1.000	1.000	1.000	1.000	1.000	1.000	1.000	1.000	1.000	1.000	1.000
**11**	0.605	0.613	0.749	0.749	0.752	0.752	0.753	0.759	0.762	0.760	0.794	0.794	0.877	0.877	0.877	0.864	0.864	0.863	1.000	1.000
**12**	0.777	0.787	0.682	0.682	0.687	0.687	0.690	0.702	0.708	0.693	0.978	0.978	0.967	0.967	0.967	0.965	0.965	0.972	1.000	1.000
**13**	0.628	0.609	0.743	0.743	0.745	0.745	0.746	0.749	0.751	0.742	0.774	0.774	0.704	0.704	0.704	0.712	0.713	1.000	1.000	1.000
**14**	0.373	0.384	0.483	0.622	0.621	1.000	1.000	1.000	1.000	1.000	1.000	1.000	1.000	1.000	1.000	1.000	1.000	1.000	1.000	1.000
**15**	-0.429	0.170	0.170	0.170	0.363	0.363	0.616	0.999	0.995	0.994	1.000	1.000	1.000	1.000	1.000	1.000	1.000	1.000	1.000	1.000
**16**	-0.363	0.173	0.175	0.175	0.416	0.416	0.717	0.999	0.999	0.999	0.999	0.999	1.000	1.000	1.000	1.000	1.000	1.000	1.000	1.000
**17**	-0.419	0.224	0.227	0.226	0.431	0.431	0.696	0.983	0.983	0.983	0.984	0.984	0.985	0.985	0.985	0.987	1.000	1.000	1.000	1.000
**18**	-0.328	0.495	0.494	0.494	0.663	0.663	1.000	1.000	1.000	1.000	1.000	1.000	1.000	1.000	1.000	1.000	1.000	1.000	1.000	1.000
**19**	-0.004	0.299	0.298	0.451	0.752	0.752	0.752	0.752	0.752	0.751	0.751	0.751	0.751	0.750	0.750	0.746	0.746	0.746	0.745	1.000
**20**	-0.371	0.660	0.659	0.660	0.988	0.988	0.988	0.988	0.988	0.988	0.988	0.988	0.988	0.988	0.988	0.989	0.989	0.989	0.989	1.000

Figure S2: Performance characteristics of different chromatin state models: Each column represents a state model for a state number between 1 and 20. Each cell represents the performance of the corresponding state model *versus* the 20-state model. Performance values are colour-coded. The optimum state model combines a minimal number of states and a maximal set of performance values. The vertical grey lines highlight the 11-state model used in this study (comprising 10 states with modified histone occupancies and 1 zero-modification state).
