# Supplementary material for: Chromatin state analysis of the barley epigenome reveals a higher‐order structure defined by H3K27me1 and H3K27me3 abundance
Source: Plant J. 2015 Sep 9;84(1):111–24. doi: 10.1111/tpj.12963 (PMC4973852; doi:10.1111/tpj.12963)
Supplement: Supplementary file 3 — Figure S3. High‐order epigenomic structures of barley chromosomes. [file TPJ-84-111-s003.pdf]

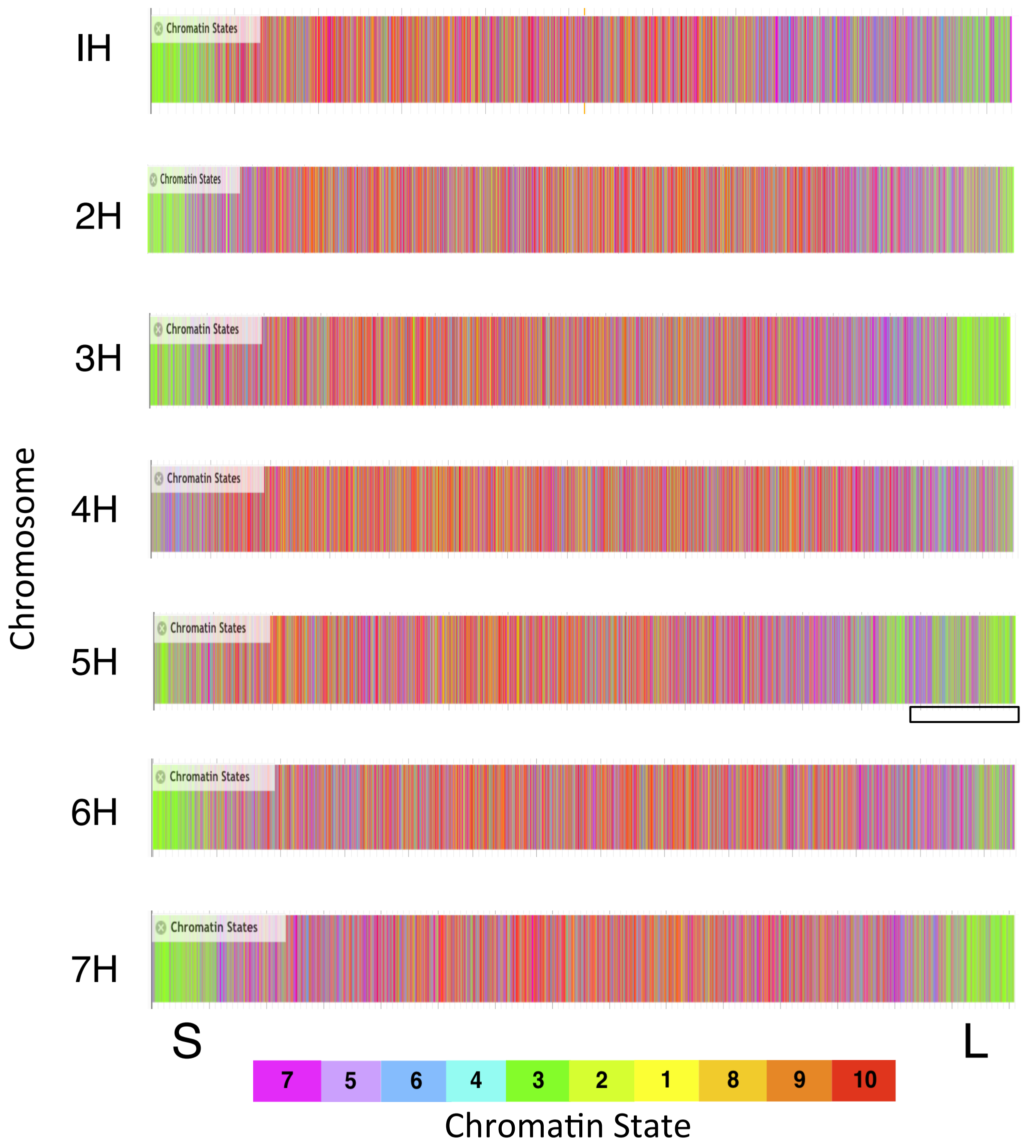

Figure S3: High-order epigenomic structures of barley chromosomes: Chromosomes (normalized to equal lengths in the figure) are visualized in JBrowse. States are colour-coded as shown. Short and long chromosome arms are indicated by S and L respectively. The box shows the region of chromosome 5HL translocated from chromosome 4HL (see Discussion).
