# Supplementary figures and images for: Chromatin state analysis of the barley epigenome reveals a higher‐order structure defined by H3K27me1 and H3K27me3 abundance
Source: Plant J. 2015 Sep 9;84(1):111–24. doi: 10.1111/tpj.12963 (PMC4973852; doi:10.1111/tpj.12963)

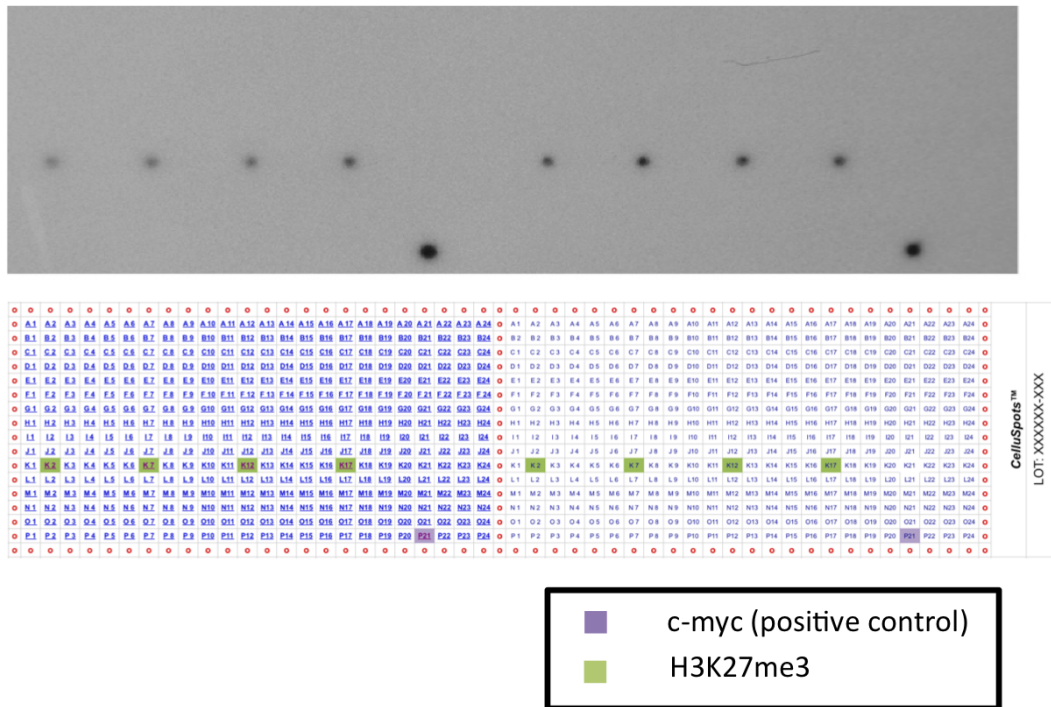

Supplement: Supplementary file 4 — Figure S4. H3K27me3 histone antibody validation. [file TPJ-84-111-s004.pdf]
