# Supplementary material for: Chromatin state analysis of the barley epigenome reveals a higher‐order structure defined by H3K27me1 and H3K27me3 abundance
Source: Plant J. 2015 Sep 9;84(1):111–24. doi: 10.1111/tpj.12963 (PMC4973852; doi:10.1111/tpj.12963)
Supplement: Supplementary file 5 — Figure S5. Relationship between distribution of H3K27me3 and H3K27me1/H3K9me2. [file TPJ-84-111-s005.pdf]

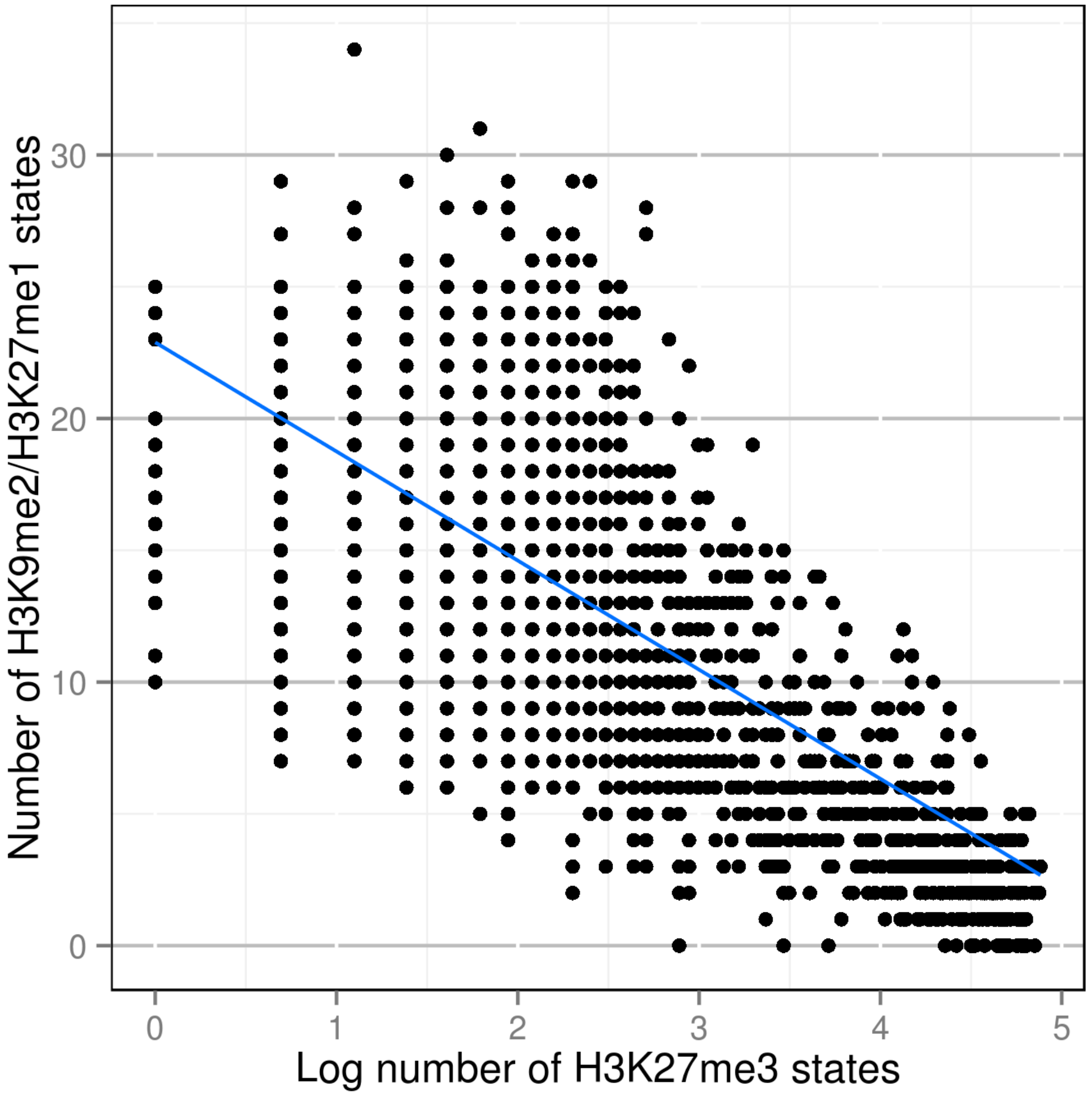

Figure S5: Relationship between distribution of H3K27me3 and H3K27me1/H3K9me2: Each spot represents a 0.5 Mbp bin containing the corresponding number of chromatin states for [H3k9me2+H3K27me1] and [H3K27me3] over the entire Barley genome. Log = natural logarithm.
