# Supplementary material for: Chromatin state analysis of the barley epigenome reveals a higher‐order structure defined by H3K27me1 and H3K27me3 abundance
Source: Plant J. 2015 Sep 9;84(1):111–24. doi: 10.1111/tpj.12963 (PMC4973852; doi:10.1111/tpj.12963)
Supplement: Supplementary file 6 — Figure S6. Average gene expression levels in chromatin states. [file TPJ-84-111-s006.pdf]

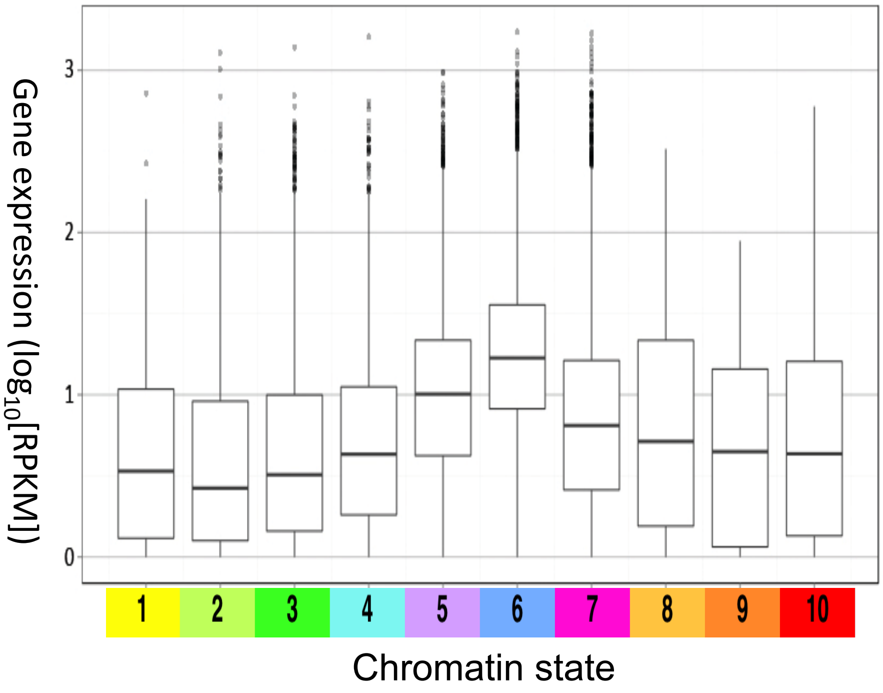

Figure S6: Average gene expression levels in chromatin states: Gene expression values are derived from RNA-seq data in total barley seedling (IBGSC 2012) and expressed as log_{10} RPKM of genes in each chromatin state (see Methods). Boxplots indicate median gene expression (central box line), interquartile range (IQR; Q3-Q1) and whiskers extend to 1.5 IQR in both directions. Points are outliers with gene expression greater than Q3 + 1.5 IQR. Chromatin states are colour-coded as in Figures 3-5.
