# Supplementary material for: Chromatin state analysis of the barley epigenome reveals a higher‐order structure defined by H3K27me1 and H3K27me3 abundance
Source: Plant J. 2015 Sep 9;84(1):111–24. doi: 10.1111/tpj.12963 (PMC4973852; doi:10.1111/tpj.12963)
Supplement: Supplementary file 7 — Figure S7. Differential gene expression in the barley genome. [file TPJ-84-111-s007.pdf]

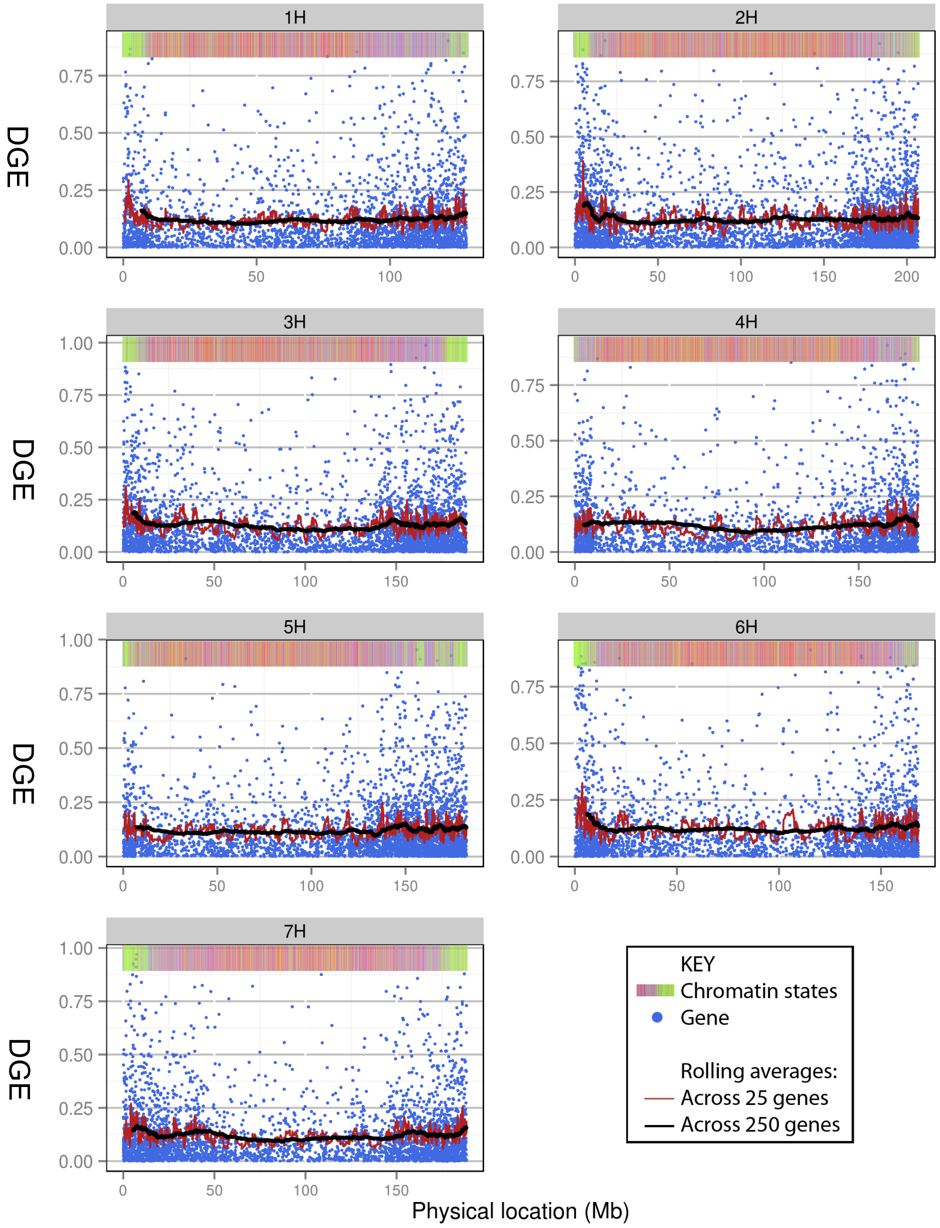

Figure S7: Differential gene expression in the barley genome: DGE was calculated for HC genes using RNA-seq seedling leaf *versus* root tissue FPKM values (IBGSC 2012). Rolling averages are plotted in windows of 25 (red lines) or 250 genes (black lines). Chromatin State designations are overlaid at the top of the plots and are colour-coded as in Figure 3-5.
