# Supplementary material for: Chromatin state analysis of the barley epigenome reveals a higher‐order structure defined by H3K27me1 and H3K27me3 abundance
Source: Plant J. 2015 Sep 9;84(1):111–24. doi: 10.1111/tpj.12963 (PMC4973852; doi:10.1111/tpj.12963)
Supplement: Supplementary file 8 — Table S1. Peak numbers for histone modifications in this study. [file TPJ-84-111-s008.pdf]

Table S1: Peak numbers for histone modifications in this study

Histone epitope	Numbers of peaks per chromosome							
	1H	2H	3H	4H	5H	6H	7H	Total
**H3K4me2**	3171	4696	4375	3385	4233	3469	4246	27575
**H3K4me3**	6625	9398	8808	6507	8788	7057	9140	56323
**H3K9me2**	1756	3098	2688	2931	2479	2257	2588	17797
**H3K9me3**	1278	2228	1973	2090	1986	1815	1970	13340
**H3K27me1**	1863	3132	2706	3045	2549	2433	2612	18340
**H3K27me2**	1300	2165	1911	2075	1870	1719	1947	12987
**H3K27me3**	2791	4147	3703	2792	4181	3053	4148	24815
**H3K36me3**	3647	5575	5391	4508	5225	4270	5032	33648
**H3K56ac**	6622	9408	8926	6678	9303	7442	10135	58514
**H3**	775	1404	1212	1291	1101	972	1133	7888
**Total**	29828	45251	41693	35302	41715	34487	42951	271227
