# Supplementary material for: Chromatin state analysis of the barley epigenome reveals a higher‐order structure defined by H3K27me1 and H3K27me3 abundance
Source: Plant J. 2015 Sep 9;84(1):111–24. doi: 10.1111/tpj.12963 (PMC4973852; doi:10.1111/tpj.12963)
Supplement: Supplementary file 9 — Table S2. Peak numbers for modified histones that are associated with genes. [file TPJ-84-111-s009.pdf]

Table S2: Peak numbers for modified histones that are associated with genes

CLASS I			
**Expression level**	**H3K4me3**	**H3K56ac**	**H3K27me3**
**zero**	180	106	228
**low**	2766	2285	1289
**mid**	3079	2915	707
**high**	3145	2958	498

CLASS II		
**Expression level**	**H3K4me2**	**H3K36me3**
**zero**	168	64
**low**	2478	1166
**mid**	2320	2099
**high**	1834	2556

CLASS III		
**Expression level**	**H3K27me2**	**H3K9me3**
**zero**	11	12
**low**	47	60
**mid**	21	56
**high**	20	45

CLASS IV		
**Expression level**	**H3K27me1**	**H3K9me2**
**zero**	7	8
**low**	37	38
**mid**	23	36
**high**	18	25

Expression level bins are defined in Methods.
