# Supplementary material for: Chromatin state analysis of the barley epigenome reveals a higher‐order structure defined by H3K27me1 and H3K27me3 abundance
Source: Plant J. 2015 Sep 9;84(1):111–24. doi: 10.1111/tpj.12963 (PMC4973852; doi:10.1111/tpj.12963)
Supplement: Supplementary file 10 — Table S3. Emissions for chromatin state models. [file TPJ-84-111-s010.pdf]

Table S3

STATE	H3K27me1	H3K27me2	H3K27me3	H3K36me3	H3K4me2	H3K4me3	H3K56ac	H3K9me2	H3K9me3	State number

state (Emission order)	H3K27me1	H3K27me2	H3K27me3	H3K36me3	H3K4me2	H3K4me3	H3K56ac	H3K9me2	H3K9me3	1
1	0.019	0.021	0.103	0.073	0.089	0.093	0.077	0.032	0.028	

state (Emission order)	H3K27me1	H3K27me2	H3K27me3	H3K36me3	H3K4me2	H3K4me3	H3K56ac	H3K9me2	H3K9me3	2
1	0.019	0.000	0.000	0.000	0.031	0.033	0.000	0.020	0.000	
2	0.018	0.374	0.295	0.416	0.020	0.027	0.308	0.026	0.357	

state (Emission order)	H3K27me1	H3K27me2	H3K27me3	H3K36me3	H3K4me2	H3K4me3	H3K56ac	H3K9me2	H3K9me3	3
1	0.031	0.020	0.000	0.000	0.000	0.000	0.000	0.033	0.019	
2	0.010	0.040	0.999	0.107	0.207	0.215	0.158	0.013	0.024	
3	0.027	0.015	0.000	0.572	0.470	0.379	0.397	0.036	0.013	

1	0.031	0.020	0.000	0.000	0.000	0.000	0.000	0.033	0.019	4
2	0.010	0.040	0.999	0.107	0.207	0.215	0.158	0.013	0.024	
3	0.009	0.014	0.000	0.000	0.505	0.456	0.552	0.009	0.012	
4	0.040	0.015	0.000	0.999	0.444	0.323	0.282	0.056	0.014	

state (Emission order)	H3K27me1	H3K27me2	H3K27me3	H3K36me3	H3K4me2	H3K4me3	H3K56ac	H3K9me2	H3K9me3	5
1	0.027	0.020	0.000	0.000	0.000	0.000	0.000	0.000	0.019	
2	0.167	0.030	0.041	0.156	0.014	0.006	0.007	0.998	0.031	
3	0.027	0.014	0.000	0.999	0.468	0.342	0.298	0.001	0.013	
4	0.008	0.014	0.000	0.000	0.506	0.458	0.555	0.001	0.012	
5	0.008	0.039	0.999	0.104	0.209	0.217	0.159	0.001	0.024	

state (Emission order)	H3K27me1	H3K27me2	H3K27me3	H3K36me3	H3K4me2	H3K4me3	H3K56ac	H3K9me2	H3K9me3	6
1	0.027	0.020	0.000	0.000	0.000	0.000	0.000	0.000	0.019	
2	0.167	0.030	0.041	0.156	0.014	0.006	0.007	0.998	0.031	
3	0.027	0.014	0.000	1.000	0.468	0.342	0.299	0.001	0.013	
4	0.011	0.017	0.000	0.000	0.999	0.283	0.001	0.001	0.012	
5	0.007	0.012	0.000	0.000	0.294	0.551	0.825	0.001	0.012	
6	0.008	0.039	0.999	0.104	0.209	0.217	0.159	0.001	0.024	

state (Emission order)	H3K27me1	H3K27me2	H3K27me3	H3K36me3	H3K4me2	H3K4me3	H3K56ac	H3K9me2	H3K9me3	7
1	0.000	0.020	0.000	0.000	0.000	0.000	0.000	0.000	0.018	
2	0.998	0.031	0.033	0.097	0.014	0.005	0.008	0.000	0.029	
3	0.167	0.030	0.041	0.156	0.014	0.006	0.007	0.999	0.031	
4	0.001	0.013	0.000	1.000	0.479	0.351	0.306	0.001	0.012	
5	0.001	0.017	0.000	0.000	0.999	0.286	0.001	0.001	0.011	
6	0.001	0.012	0.000	0.000	0.295	0.552	0.827	0.001	0.012	
7	0.000	0.038	0.999	0.103	0.210	0.219	0.160	0.001	0.023	

state (Emission order)	H3K27me1	H3K27me2	H3K27me3	H3K36me3	H3K4me2	H3K4me3	H3K56ac	H3K9me2	H3K9me3	8
1	0.167	0.030	0.041	0.156	0.014	0.006	0.007	0.999	0.031	
2	0.998	0.029	0.032	0.097	0.014	0.005	0.008	0.000	0.028	
3	0.000	0.000	0.000	0.000	0.000	0.000	0.000	0.000	0.017	
4	0.003	0.998	0.198	0.074	0.041	0.023	0.044	0.000	0.077	
5	0.001	0.000	0.999	0.102	0.215	0.225	0.161	0.001	0.020	
6	0.001	0.000	0.000	0.000	0.999	0.289	0.001	0.001	0.010	
7	0.001	0.001	0.000	1.000	0.484	0.355	0.310	0.001	0.011	
8	0.001	0.001	0.000	0.000	0.298	0.555	0.828	0.001	0.011	

state (Emission order)	H3K27me1	H3K27me2	H3K27me3	H3K36me3	H3K4me2	H3K4me3	H3K56ac	H3K9me2	H3K9me3	9
1	0.998	0.029	0.032	0.097	0.014	0.005	0.008	0.000	0.028	
2	0.167	0.030	0.041	0.156	0.014	0.006	0.007	0.999	0.031	
3	0.000	0.000	0.000	0.000	0.000	0.000	0.000	0.000	0.000	
4	0.001	0.001	0.000	1.000	0.488	0.357	0.312	0.001	0.003	
5	0.001	0.000	0.000	0.000	0.999	0.291	0.000	0.001	0.002	
6	0.001	0.001	0.000	0.000	0.300	0.557	0.829	0.001	0.003	
7	0.001	0.000	0.999	0.102	0.218	0.228	0.162	0.001	0.004	
8	0.003	0.999	0.198	0.074	0.041	0.023	0.045	0.000	0.077	
9	0.000	0.000	0.107	0.052	0.019	0.010	0.028	0.000	0.999	

state (Emission order)	H3K27me1	H3K27me2	H3K27me3	H3K36me3	H3K4me2	H3K4me3	H3K56ac	H3K9me2	H3K9me3	10
1	0.999	0.029	0.032	0.094	0.011	0.005	0.008	0.000	0.028	
2	0.167	0.030	0.041	0.154	0.012	0.006	0.007	0.999	0.031	
3	0.001	0.000	0.000	0.999	0.000	0.268	0.266	0.001	0.001	
4	0.003	0.003	0.074	1.000	1.000	0.465	0.362	0.004	0.007	
5	0.001	0.000	0.000	0.000	0.999	0.291	0.001	0.001	0.002	

**6**	0.001	0.001	0.000	0.000	0.300	0.557	0.830	0.001	0.003
**7**	0.000	0.000	0.000	0.000	0.000	0.000	0.000	0.000	0.000
**8**	0.000	0.000	0.999	0.075	0.194	0.214	0.155	0.001	0.003
**9**	0.003	0.999	0.196	0.070	0.037	0.021	0.043	0.000	0.077
**10**	0.000	0.000	0.108	0.051	0.016	0.011	0.029	0.000	0.999

**state (Emission order)**	**H3K27me1**	**H3K27me2**	**H3K27me3**	**H3K36me3**	**H3K4me2**	**H3K4me3**	**H3K56ac**	**H3K9me2**	**H3K9me3**	**11**
**1**	0.003	0.999	0.186	0.069	0.027	0.011	0.036	0.000	0.077	
**2**	0.000	0.000	0.999	0.081	0.000	0.000	0.098	0.000	0.022	
**3**	0.003	0.010	0.999	0.154	0.708	0.737	0.308	0.005	0.016	
**4**	0.001	0.001	0.000	0.000	0.999	0.292	0.001	0.001	0.002	
**5**	0.004	0.002	0.000	1.000	1.000	0.449	0.358	0.003	0.006	
**6**	0.001	0.000	0.000	0.999	0.000	0.269	0.266	0.001	0.001	
**11 (7)**	0.000	0.000	0.000	0.000	0.000	0.000	0.000	0.000	0.000	
**7 (8)**	0.001	0.001	0.000	0.000	0.300	0.558	0.830	0.001	0.004	
**8 (9)**	0.000	0.000	0.001	0.043	0.012	0.008	0.018	0.000	0.999	
**9 (10)**	0.999	0.029	0.031	0.094	0.010	0.005	0.008	0.000	0.028	
**10 (11)**	0.167	0.030	0.040	0.154	0.012	0.005	0.007	0.999	0.031	

For the 11-state model that was chosen for this study the states were reordered in the manuscript to place the zero state (no associated histone peaks) last (i.e. 7->11). Numbers in brackets are the original state numbers outputted by ChromHMM.

**state (Emission order)**	**H3K27me1**	**H3K27me2**	**H3K27me3**	**H3K36me3**	**H3K4me2**	**H3K4me3**	**H3K56ac**	**H3K9me2**	**H3K9me3**	**12**
**1**	0.003	0.999	0.186	0.069	0.027	0.011	0.035	0.000	0.077	
**2**	0.000	0.000	0.999	0.081	0.000	0.000	0.098	0.000	0.022	
**3**	0.003	0.010	0.999	0.154	0.708	0.737	0.308	0.005	0.016	
**4**	0.001	0.001	0.000	0.000	0.999	0.297	0.001	0.002	0.002	
**5**	0.004	0.002	0.000	1.000	1.000	0.449	0.358	0.003	0.006	
**6**	0.001	0.000	0.000	0.999	0.000	0.268	0.266	0.001	0.001	
**7**	0.000	0.000	0.000	0.000	0.000	0.995	0.000	0.000	0.000	
**8**	0.002	0.002	0.000	0.000	0.350	0.490	0.998	0.001	0.004	
**9**	0.000	0.000	0.000	0.000	0.000	0.000	0.000	0.000	0.000	
**10**	0.000	0.000	0.001	0.043	0.012	0.008	0.017	0.000	0.999	
**11**	0.999	0.029	0.031	0.094	0.010	0.005	0.008	0.000	0.028	
**12**	0.167	0.030	0.040	0.154	0.011	0.005	0.007	0.999	0.031	

**state (Emission order)**	**H3K27me1**	**H3K27me2**	**H3K27me3**	**H3K36me3**	**H3K4me2**	**H3K4me3**	**H3K56ac**	**H3K9me2**	**H3K9me3**	**13**
**1**	0.002	0.002	0.000	0.000	0.350	0.490	0.998	0.002	0.004	
**2**	0.000	0.000	0.000	0.000	0.000	0.000	0.000	0.000	0.000	
**3**	0.004	0.039	0.999	0.124	0.148	0.000	0.995	0.006	0.028	
**4**	0.000	0.000	0.999	0.085	0.087	0.000	0.000	0.000	0.021	
**5**	0.002	0.011	0.999	0.151	0.628	0.989	0.348	0.004	0.015	
**6**	0.000	0.000	0.000	0.000	0.000	0.995	0.000	0.000	0.000	
**7**	0.001	0.001	0.000	0.000	0.999	0.297	0.001	0.001	0.002	
**8**	0.003	0.002	0.000	1.000	1.000	0.449	0.359	0.002	0.006	
**9**	0.001	0.000	0.000	0.999	0.000	0.268	0.266	0.001	0.001	
**10**	0.167	0.030	0.040	0.154	0.013	0.005	0.006	0.999	0.031	
**11**	0.999	0.030	0.032	0.094	0.010	0.005	0.007	0.000	0.029	
**12**	0.000	0.000	0.001	0.043	0.012	0.008	0.017	0.000	0.999	
**13**	0.001	0.999	0.174	0.067	0.028	0.009	0.021	0.000	0.076	

**state (Emission order)**	**H3K27me1**	**H3K27me2**	**H3K27me3**	**H3K36me3**	**H3K4me2**	**H3K4me3**	**H3K56ac**	**H3K9me2**	**H3K9me3**	**14**
**1**	0.999	0.030	0.032	0.093	0.010	0.005	0.007	0.000	0.029	
**2**	0.167	0.030	0.040	0.153	0.011	0.005	0.006	0.999	0.031	
**3**	0.001	0.000	0.000	0.999	0.000	0.268	0.266	0.001	0.001	
**4**	0.003	0.002	0.000	1.000	0.999	0.998	0.607	0.003	0.006	
**5**	0.005	0.004	0.000	1.000	1.000	0.000	0.154	0.006	0.007	
**6**	0.001	0.001	0.000	0.000	0.999	0.297	0.001	0.002	0.002	
**7**	0.002	0.011	0.999	0.151	0.628	0.989	0.348	0.004	0.015	
**8**	0.000	0.000	0.999	0.085	0.087	0.000	0.000	0.000	0.021	
**9**	0.004	0.039	0.999	0.124	0.148	0.000	0.995	0.006	0.028	
**10**	0.000	0.000	0.000	0.000	0.000	0.000	0.000	0.000	0.000	
**11**	0.002	0.002	0.000	0.000	0.350	0.490	0.998	0.002	0.004	
**12**	0.000	0.000	0.000	0.000	0.000	0.995	0.000	0.000	0.000	
**13**	0.000	0.000	0.001	0.043	0.012	0.008	0.017	0.000	0.999	
**14**	0.001	0.999	0.174	0.065	0.026	0.009	0.021	0.000	0.076	

**state (Emission order)**	H3K27me1	H3K27me2	H3K27me3	H3K36me3	H3K4me2	H3K4me3	H3K56ac	H3K9me2	H3K9me3	**15**
**1**	0.999	0.030	0.032	0.093	0.010	0.005	0.007	0.000	0.029	
**2**	0.167	0.030	0.040	0.152	0.011	0.005	0.006	0.999	0.031	
**3**	0.000	0.000	0.000	0.999	0.000	0.000	0.085	0.000	0.000	
**4**	0.005	0.004	0.000	1.000	1.000	0.000	0.154	0.006	0.007	
**5**	0.003	0.002	0.000	1.000	1.000	0.998	0.607	0.003	0.006	
**6**	0.002	0.001	0.000	1.000	0.000	0.991	0.751	0.002	0.003	
**7**	0.000	0.000	0.000	0.000	0.000	0.995	0.000	0.000	0.000	
**8**	0.002	0.011	0.999	0.151	0.628	0.989	0.348	0.004	0.015	
**9**	0.000	0.000	0.999	0.085	0.087	0.000	0.000	0.000	0.021	
**10**	0.004	0.039	0.999	0.124	0.148	0.000	0.995	0.006	0.028	
**11**	0.000	0.000	0.000	0.000	0.000	0.000	0.000	0.000	0.000	
**12**	0.002	0.002	0.000	0.000	0.350	0.490	0.998	0.002	0.004	
**13**	0.001	0.001	0.000	0.000	0.999	0.297	0.001	0.002	0.002	

14	0.000	0.000	0.001	0.044	0.012	0.008	0.018	0.000	0.999	
15	0.001	0.999	0.174	0.065	0.026	0.009	0.020	0.000	0.076	

state (Emission order)	H3K27me1	H3K27me2	H3K27me3	H3K36me3	H3K4me2	H3K4me3	H3K56ac	H3K9me2	H3K9me3	16
1	0.002	0.002	0.000	0.000	0.350	0.490	0.998	0.002	0.004	
2	0.000	0.000	0.000	0.000	0.000	0.000	0.000	0.000	0.000	
3	0.002	0.035	0.999	0.000	0.137	0.001	0.995	0.004	0.026	
4	0.000	0.000	0.999	0.000	0.081	0.000	0.000	0.000	0.021	
5	0.015	0.035	0.999	0.999	0.299	0.312	0.242	0.029	0.029	
6	0.000	0.000	0.000	0.999	0.000	0.000	0.085	0.000	0.000	
7	0.005	0.004	0.000	1.000	1.000	0.000	0.154	0.006	0.007	
8	0.003	0.002	0.000	1.000	1.000	0.998	0.607	0.003	0.006	
9	0.002	0.001	0.000	1.000	0.000	0.991	0.751	0.003	0.003	
10	0.000	0.000	0.000	0.000	0.000	0.995	0.000	0.000	0.000	
11	0.002	0.009	0.999	0.000	0.631	0.988	0.329	0.003	0.014	
12	0.001	0.001	0.000	0.000	0.999	0.297	0.001	0.002	0.002	
13	0.000	0.000	0.001	0.044	0.012	0.008	0.018	0.000	0.999	
14	0.001	0.999	0.166	0.055	0.024	0.008	0.021	0.000	0.077	
15	0.999	0.029	0.027	0.088	0.010	0.005	0.007	0.000	0.028	
16	0.169	0.029	0.032	0.145	0.010	0.005	0.006	0.999	0.031	

state (Emission order)	H3K27me1	H3K27me2	H3K27me3	H3K36me3	H3K4me2	H3K4me3	H3K56ac	H3K9me2	H3K9me3	17
1	0.001	0.001	0.000	0.000	0.999	0.295	0.006	0.002	0.002	
2	0.005	0.004	0.000	1.000	1.000	0.000	0.155	0.006	0.007	
3	0.003	0.002	0.000	1.000	1.000	0.998	0.607	0.003	0.006	
4	0.002	0.001	0.000	1.000	0.000	0.996	0.750	0.003	0.003	
5	0.000	0.000	0.000	0.999	0.000	0.000	0.086	0.000	0.000	
6	0.015	0.035	0.999	0.999	0.299	0.312	0.242	0.029	0.029	
7	0.000	0.000	0.999	0.000	0.081	0.000	0.000	0.000	0.021	
8	0.002	0.035	0.999	0.000	0.137	0.000	0.995	0.004	0.026	
9	0.001	0.000	0.000	0.000	0.171	0.000	0.996	0.001	0.001	
10	0.002	0.003	0.000	0.000	0.529	0.998	0.999	0.002	0.007	
11	0.000	0.000	0.000	0.000	0.000	0.995	0.000	0.000	0.000	
12	0.002	0.009	0.999	0.000	0.631	0.988	0.329	0.003	0.014	
13	0.001	0.999	0.166	0.055	0.025	0.008	0.021	0.000	0.077	
14	0.000	0.000	0.000	0.000	0.000	0.000	0.000	0.000	0.000	
15	0.999	0.029	0.027	0.088	0.010	0.005	0.007	0.000	0.028	
16	0.169	0.029	0.032	0.145	0.010	0.005	0.006	0.999	0.031	
17	0.000	0.000	0.001	0.044	0.013	0.008	0.019	0.000	0.999	

state (Emission order)	H3K27me1	H3K27me2	H3K27me3	H3K36me3	H3K4me2	H3K4me3	H3K56ac	H3K9me2	H3K9me3	18
1	0.002	0.001	0.000	1.000	0.000	0.996	0.750	0.003	0.003	
2	0.003	0.002	0.000	1.000	1.000	0.998	0.607	0.003	0.006	
3	0.006	0.004	0.000	1.000	1.000	0.000	0.154	0.006	0.007	
4	0.000	0.000	0.000	0.999	0.000	0.000	0.086	0.000	0.000	
5	0.023	0.056	0.999	0.998	0.166	0.177	0.182	0.040	0.035	
6	0.000	0.000	0.999	0.000	0.000	0.000	0.000	0.000	0.021	
7	0.002	0.036	0.999	0.000	0.000	0.002	0.995	0.005	0.026	
8	0.001	0.000	0.000	0.000	0.171	0.000	0.996	0.001	0.001	
9	0.002	0.003	0.000	0.000	0.529	0.998	0.999	0.002	0.007	
10	0.000	0.000	0.000	0.000	0.000	0.995	0.000	0.000	0.000	
11	0.002	0.008	0.999	0.084	0.658	0.998	0.347	0.003	0.014	
12	0.006	0.025	0.999	0.002	0.998	0.000	0.165	0.007	0.019	
13	0.010	0.002	0.000	0.000	0.999	0.293	0.007	0.002	0.003	
14	0.000	0.000	0.001	0.044	0.012	0.008	0.019	0.000	0.999	
15	0.006	0.999	0.156	0.049	0.019	0.009	0.021	0.000	0.076	
16	0.000	0.000	0.000	0.000	0.000	0.000	0.000	0.000	0.000	
17	0.999	0.026	0.023	0.088	0.001	0.004	0.007	0.000	0.027	
18	0.168	0.029	0.029	0.144	0.009	0.005	0.006	0.999	0.030	

state (Emission order)	H3K27me1	H3K27me2	H3K27me3	H3K36me3	H3K4me2	H3K4me3	H3K56ac	H3K9me2	H3K9me3	19
1	0.002	0.002	0.000	1.000	0.000	0.996	0.750	0.003	0.003	
2	0.003	0.002	0.000	1.000	1.000	0.999	0.607	0.003	0.006	
3	0.006	0.004	0.000	1.000	1.000	0.000	0.154	0.006	0.007	
4	0.000	0.000	0.000	0.999	0.000	0.000	0.086	0.000	0.000	
5	0.028	0.063	0.999	0.998	0.152	0.007	0.138	0.047	0.038	
6	0.000	0.000	0.999	0.000	0.000	0.000	0.000	0.000	0.021	
7	0.002	0.036	0.999	0.001	0.000	0.000	0.995	0.005	0.026	
8	0.001	0.000	0.000	0.000	0.170	0.000	0.996	0.001	0.001	
9	0.002	0.003	0.000	0.000	0.529	0.998	0.999	0.002	0.007	
10	0.000	0.000	0.000	0.000	0.000	0.995	0.000	0.000	0.000	
11	0.002	0.014	0.999	0.154	0.000	0.996	0.324	0.003	0.016	
12	0.002	0.011	1.000	0.146	1.000	0.998	0.365	0.005	0.015	
13	0.006	0.025	0.999	0.003	0.998	0.000	0.165	0.007	0.019	
14	0.010	0.002	0.000	0.000	0.999	0.293	0.008	0.002	0.003	
15	0.000	0.000	0.001	0.044	0.012	0.008	0.019	0.000	0.999	
16	0.006	0.999	0.154	0.048	0.019	0.007	0.021	0.000	0.076	
17	0.000	0.000	0.000	0.000	0.000	0.000	0.000	0.000	0.000	
18	0.999	0.025	0.022	0.088	0.001	0.004	0.007	0.000	0.027	
19	0.168	0.029	0.028	0.143	0.009	0.005	0.006	0.999	0.030	

state (Emission order)	H3K27me1	H3K27me2	H3K27me3	H3K36me3	H3K4me2	H3K4me3	H3K56ac	H3K9me2	H3K9me3	20
1	0.002	0.002	0.000	1.000	0.000	0.996	0.751	0.002	0.003	
2	0.002	0.002	0.000	1.000	1.000	0.998	0.607	0.003	0.006	

**3**	0.006	0.004	0.000	1.000	1.000	0.000	0.155	0.000	0.007
**4**	0.000	0.000	0.000	0.999	0.000	0.000	0.086	0.000	0.000
**5**	0.023	0.056	0.999	0.999	0.157	0.002	0.142	0.001	0.035
**6**	0.000	0.000	0.999	0.000	0.000	0.000	0.000	0.000	0.021
**7**	0.002	0.036	0.999	0.001	0.000	0.000	0.995	0.005	0.026
**8**	0.001	0.000	0.000	0.000	0.170	0.000	0.996	0.001	0.001
**9**	0.002	0.003	0.000	0.000	0.529	0.998	0.999	0.002	0.007
**10**	0.000	0.000	0.000	0.000	0.000	0.995	0.000	0.000	0.000
**11**	0.003	0.014	0.999	0.156	0.000	0.996	0.324	0.003	0.016
**12**	0.002	0.011	1.000	0.147	1.000	0.998	0.365	0.005	0.015
**13**	0.006	0.025	0.999	0.002	0.998	0.000	0.165	0.007	0.019
**14**	0.010	0.002	0.000	0.000	0.999	0.293	0.008	0.002	0.003
**15**	0.000	0.000	0.001	0.044	0.012	0.008	0.019	0.000	0.999
**16**	0.007	0.999	0.155	0.048	0.019	0.007	0.021	0.000	0.076
**17**	0.000	0.000	0.000	0.000	0.000	0.000	0.000	0.000	0.000
**18**	0.999	0.025	0.022	0.087	0.000	0.004	0.007	0.000	0.027
**19**	0.248	0.049	0.077	0.999	0.040	0.010	0.015	0.999	0.040
**20**	0.152	0.027	0.032	0.000	0.008	0.005	0.005	0.999	0.029
