# Supplementary material for: Chromatin state analysis of the barley epigenome reveals a higher‐order structure defined by H3K27me1 and H3K27me3 abundance
Source: Plant J. 2015 Sep 9;84(1):111–24. doi: 10.1111/tpj.12963 (PMC4973852; doi:10.1111/tpj.12963)
Supplement: Supplementary file 11 — Table S4. Antibodies used in this study. [file TPJ-84-111-s011.pdf]

**Table S4: Antibodies used in this study**

| Targeted epitope | Clonality | Manufacturer | Product code | Lot number |
|---|---|---|---|---|
| H3 | Polyclonal | Abcam | ab1791 | GR81366-1 |
| H3K4me2 | Monoclonal | Abcam | ab32356 | GR39894-3 |
| H3K4me3 | Polyclonal | Active Motif | 39159 | 1609004 |
| H3K9me2 | Polyclonal | Active Motif | 39375 | 29108001 |
| H3K9me3 | Polyclonal | Millipore | 07-442 | JBC1865906 |
| H3K27me1 | Polyclonal | Millipore | 07-448 | DAM1661077 |
| H3K27me2 | Polyclonal | Millipore | 07-452 | JBC1870071 |
| H3K27me3 | Polyclonal | Millipore | 07-449 | JBC1873477 |
| H3K36me3 | Polyclonal | Abcam | ab9050 | GR20245-2 |
| H3K56ac | Polyclonal | Active Motif | 39281 | 16908001 |
