# Supplementary material for: Chromatin state analysis of the barley epigenome reveals a higher‐order structure defined by H3K27me1 and H3K27me3 abundance
Source: Plant J. 2015 Sep 9;84(1):111–24. doi: 10.1111/tpj.12963 (PMC4973852; doi:10.1111/tpj.12963)
Supplement: Supplementary file 12 — Table S5. Quantitative PCR validation of peak finding software. [file TPJ-84-111-s012.pdf]

## Table S5: qPCR validation of peak finding softwares

| Gene | | Forward primer (5' → 3') | Reverse primer (5' → 3') | H3K56 peak identification qPCR | CCAT | FindPeaks | SISSRS |
|---|---|---|---|---|---|---|---|
| *DREB1* | MLOC_66686 | CTTTGTCCCCTATCCCTTGTC | CAACTCCCATTGACGCATAA | −++ | −−+ | −−− | −−− |
| *HKT1* | MLOC_13204 | GAGTGACGGAGGGAAGTTTG | CAATTTGGTACATTTGCCTTTC | −−+ | −++ | −−+ | −−− |
| *HVA* | MLOC_59546 | TGGGGACTGGATAAGAAACTG | AGCCAATTAACAGATGGGAAAT | −++ | −−+ | −−− | −−− |
| *HVP10* | MLOC_36965 | GGATTGCCGCAGTTACCTAC | TACCAACGAGGGAGGACATC | −−− | −+− | −+− | −−− |
| *NHX1* | MLOC_4602 | GCATATTGACAAAGCCTCTGAT | GAGGAGAGTGCAGGGACTTC | +−− | −+− | −−− | −−− |
| *RAF* | MLOC_13305 | CCCGAGCTGATGGAGTTTT | CGCTCCCCACAGAAGAGTC | +−+ | +++ | +−− | −+− |
| *SOS1* | MLOC_36509 | CAAGGATTTTGCTCCCTCAG | ATGCCCGAGTTCAATTTCTT | −−− | −−− | −−− | −−− |
| *SOS2* | MLOC_67101 | GAAGTGGGACGACGACAAC | CTCGAACAGCCCCGATAG | +++ | +++ | ++− | −−− |
| | | | | **Accuracy** | ***70%*** | ***63%*** | ***48%*** |
