# Supplementary material for: Chromatin state analysis of the barley epigenome reveals a higher‐order structure defined by H3K27me1 and H3K27me3 abundance
Source: Plant J. 2015 Sep 9;84(1):111–24. doi: 10.1111/tpj.12963 (PMC4973852; doi:10.1111/tpj.12963)
Supplement: Supplementary file 13 [file TPJ-84-111-s013.docx]

**SUPPORTING INFORMATION**

**Figure S1: Peak densities of histone modifications across barley chromosomes:** Local peak densities for the nine histone modifications studied here are plotted against pseudo-physical positions on barley chromosomes in JBrowse (see Methods). Distribution Classes I-IV are described in the text. The distributions of HC genes, TEs and histone H3 are also shown and the location of the LR-PC region ([Baker *et al.* 2014](#_ENREF_1)) is indicated in grey.

**Figure S2: Performance characteristics of different chromatin state models:** Each column represents a state model for a state number between 1 and 20. Each cell represents the performance of the corresponding state model *versus* the 20-state model. Performance values are colour-coded. The optimum state model combines a minimal number of states and a maximal set of performance values. The vertical grey lines highlight the 11-state model used in this study (comprising 10 states with modified histone occupancies and 1 zero-modification state).

**Figure S3: High-order epigenomic structures of barley chromosomes:** Chromosomes (normalized to equal lengths in the figure) are visualized in JBrowse. States are colour-coded as shown. Short and long chromosome arms are indicated by S and L respectively. The box shows the region of chromosome 5HL translocated from chromosome 4HL (see Discussion).

**Figure S4: H3K27me3 histone antibody validation:** The H3K27me3 antibody batch used in this study was checked by binding to the Active Motif MODified Histone Peptide Array (cat.no. 13005) using the manufacturers protocol. Green sectors in the key refer to peptides containing H3K27me3. Purple sectors refer to peptides containing c-myc positive control.

**Figure S5: Relationship between distribution of H3K27me3 and H3K27me1/H3K9me2:** Each spot represents a 0.5 Mbp bin containing the corresponding number of chromatin states for [H3k9me2+H3K27me1] and [H3K27me3] over the entire Barley genome. Log = natural logarithm.

**Figure S6: Average gene expression levels in chromatin states:** Gene expression values are derived from RNA-seq data in total barley seedling ([IBGSC 2012](#_ENREF_16)) and expressed as log_10_ RPKM of genes in each chromatin state (see Methods). Boxplots indicate median gene expression (central box line), interquartile range (IQR; Q3-Q1) and whiskers extend to 1.5 IQR in both directions. Points are outliers with gene expression greater than Q3 + 1.5 IQR. Chromatin states are colour-coded as in Figures 3-5.

**Figure S7: Differential gene expression in the barley genome:** DGE was calculated for HC genes using RNA-seq seedling leaf *versus* root tissue FPKM values ([IBGSC 2012](#_ENREF_16)). Rolling averages are plotted in windows of 25 (red lines) or 250 genes (black lines). Chromatin State designations are overlaid at the top of the plots and are colour-coded as in Figure 3-5.

**Table S1: Peak numbers for histone modifications in this study:** Peaks were called from ChIP-seq data using CCAT (see Methods).

**Table S2: Peak numbers for modified histones that are associated with genes:** Expression level bins are defined in Methods.

**Table S3: Emissions for chromatin state models:** State emissions for state models between 1 and 20 for the barley epigenome derived from peak sharing among the 9 histone modifications in this study. For each state model the states (first column) are represented by rows. Histone modifications (columns B-J) are ordered alphabetically. Each cell represents the emission for the corresponding modification in the corresponding state.

For the 11-state model that was chosen for this study the states were reordered in the manuscript to place the zero state (no associated histone peaks) last (i.e. 7->11). Numbers in brackets are the original state numbers outputted by ChromHMM.

**Table S4: Antibodies used in this study**

**Table S5: qPCR validation of peak finding softwares:** 8 genes were scored in triplicate for H3K56ac peaks, either from ChIP-seq data using three different softwares or from independent qPCR from chromatin preparations. – and + symbols refer to whether a peak was called by the corresponding software or qPCR respectively. Each symbol refers to a biological replicate. Accuracy values refer to the percentage agreement between the summed results over all 8 genes analysed for the corresponding peak finding software and the qPCR experiment.
